# Reprogramming of cancer-associated fibroblasts by apoptotic cancer cells inhibits lung metastasis via Notch1-WISP-1 signaling

**DOI:** 10.1038/s41423-022-00930-w

**Published:** 2022-10-14

**Authors:** Hee Ja Kim, Kyungwon Yang, Kiyoon Kim, Ye‐Ji Lee, Sieun Lee, Sung Yong Ahn, Young‐Ho Ahn, Jihee Lee Kang

**Affiliations:** 1 Department of Physiology, College of Medicine, Ewha Womans University, Seoul, 07804 Korea; 2 Inflammation-Cancer Microenvironment Research Center, College of Medicine, Ewha Womans University, Seoul, 07804 Korea; 3 Department of Molecular Medicine, College of Medicine, Ewha Womans University, Seoul, 07804 Korea

**Keywords:** CAFs, Apoptotic lung cancer cells, Notch1, WISP-1, Efferocytosis, Migration, Invasion, Metastasis, Cell death and immune response, Cancer microenvironment

## Abstract

The interplay between apoptotic cancer cells and the tumor microenvironment modulates cancer progression and metastasis. Cancer-associated fibroblasts (CAFs) play a crucial role in promoting these events through paracrine communication. Here, we demonstrate that conditioned medium (CM) from lung CAFs exposed to apoptotic cancer cells suppresses TGF-β1-induced migration and invasion of cancer cells and CAFs. Direct exposure of CAFs to apoptotic 344SQ cells (ApoSQ) inhibited CAF migration and invasion and the expression of CAF activation markers. Enhanced secretion of Wnt‐induced signaling protein 1 (WISP-1) by CAFs exposed to ApoSQ was required for these antimigratory and anti-invasive effects. Pharmacological inhibition of Notch1 activation or siRNA-mediated Notch1 silencing prevented WISP-1 production by CAFs and reversed the antimigratory and anti-invasive effects. Enhanced expression of the Notch ligand delta-like protein 1 on the surface of ultraviolet-irradiated apoptotic lung cancer cells triggered Notch1-WISP-1 signaling. Phosphatidylserine receptor brain-specific angiogenesis inhibitor 1 (BAI1)-Rac1 signaling, which facilitated efferocytosis by CAFs, participated in crosstalk with Notch1 signaling for optimal production of WISP-1. In addition, a single injection of ApoSQ enhanced WISP-1 production, suppressed the expression of CAF activation markers in isolated Thy1^+^ CAFs, and inhibited lung metastasis in syngeneic immunocompetent mice via Notch1 signaling. Treatment with CM from CAFs exposed to ApoSQ suppressed tumor growth and lung metastasis, whereas treatment with WISP-1-immunodepleted CM from CAFs exposed to ApoSQ reversed the antitumorigenic and antimetastatic effects. Therefore, treatment with CM from CAFs exposed to apoptotic lung cancer cells could be therapeutically applied to suppress CAF activation, thereby preventing cancer progression and metastasis.

## Introduction

Lung cancer is the most common type of cancer and the leading cause of cancer-related death worldwide, accounting for 18% of total cancer deaths [1]. Almost 75% of patients with lung cancer present with locally advanced or metastatic disease at the time of diagnosis [2, 3]. Metastasis is a multistep process that involves the migration and invasion of cancer cells, which are hallmarks of malignancy.

Carcinoma-associated fibroblasts (CAFs) with diverse origins are among the predominant cell types present within the tumor-associated stroma [4]. Paracrine communication between CAFs and cancer cells facilitates processes such as cancer cell migration and invasion, which can promote tumor progression to malignancy and metastatic spread [5]. CAFs physically remodel the matrix in the tumor stroma, allowing cancer cells to invade while still maintaining their epithelial properties [6, 7]. However, the molecular mechanisms by which CAFs control tumor progression remain unclear.

The importance of Notch signaling in regulating fibroblast activation in the tumor microenvironment (TME) is well established. Activation of Notch signaling is normally tightly controlled by direct interactions with ligand-expressing cells, and dysregulated Notch signaling is associated with developmental abnormalities and cancer. Interestingly, Notch activity is linked to both oncogenic and tumor-suppressive functions [8, 9] that are highly context dependent [10]. In addition, stromal fibroblasts with constitutively activated Notch signaling attenuate melanoma growth and suppress tumor angiogenesis partially through upregulating Wnt‐induced signaling protein 1 (WISP-1) [11]. These findings warrant further research to elucidate the molecular mechanisms underlying the regulatory role of Notch1 in CAFs in other cancer types.

High levels of cell death within the TME and clearance of dying tumor cells profoundly influence tumor-specific immunity [12]. In the TME, the immunosuppressive effect of phagocyte-mediated clearance appears to inhibit the anti-tumor immune response [13, 14]. However, tumor cells can evade immune surveillance by preventing their recognition for efferocytosis [15, 16]. In addition, anti-inflammatory and proresolving lipid autacoids inhibit debris-stimulated progression of cancer by enhancing the clearance of debris via macrophage phagocytosis in multiple tumor types [17]. Importantly, our previous studies demonstrated that macrophages exposed to ultraviolet (UV)-irradiated apoptotic lung cancer cells inhibit the polarity disruption, epithelial-to-mesenchymal transition (EMT), and invasion of cancer cells [18]. However, whether efferocytosis of cancer cells by CAFs controls the activation of CAFs in the TME and prevents cancer progression and metastasis has not been studied. Here, we investigated how the interaction of CAFs with apoptotic lung cancer cells modulates the migration and invasion of cancer cells and CAFs. We also investigated whether and how a single injection of apoptotic lung cancer cells inhibits CAF activation and lung metastasis in syngeneic immunocompetent mice. Furthermore, conditioned medium (CM) collected from CAFs exposed to apoptotic lung cancer cells was administered via intratumoral injection to evaluate its inhibitory effects on tumor progression and lung metastasis.

## Materials and methods

### Reagents

DAPT (D5942) was purchased from Sigma‒Aldrich (St. Louis, MO, USA). TGF-β1 (240-B-010) and mouse rWISP-1 (1680-WS) were purchased from R&D Systems (Minneapolis, MN, USA). LY3039478 (HY-12449) was purchased from MedChemExpress (Monmouth Junction, NJ, USA).

### Antibodies

The antibodies used for western blotting, immunofluorescence, flow cytometry, and cell sorting are listed in Table S1.

### Isolation of CAFs and cell culture

CAFs were isolated from lung tumors of Kras-mutant (*Kras*LA1) mice using magnetic-activated cell sorting with a fibroblast-specific marker, Thy1, as described previously [19]. CAFs were cultured in alpha-MEM (Welgene, Gyeongsan, Korea) supplemented with 10% fetal bovine serum (FBS; Gibco, Waltham, MA, USA), penicillin/streptomycin (100 U/100 μg, HyClone, Logan, UT, USA), 2 mM L-glutamine (Welgene), and 1 mM sodium pyruvate (Welgene). For immortalization, CAFs were stably transfected with the TERT plasmid (pCDH-3xFLAG-TERT, a gift from Steven Artandi; Addgene 51 plasmid # 51631) using Lipofector-EXT (AptaBio, Yongin, Korea). The primary cells used in the experiments were passaged fewer than six times and were not tested for identity. Human cancer cell lines were obtained from ATCC (American Type Culture Collection; Manassas, VA, USA). 344SQ cells (a gift from Dr. Jonathan M. Kurie, University of Texas MD Anderson Cancer Center, USA) and various human cancer cell lines [A549 (lung) and HCT116 (colon)] were maintained in RPMI 1640 medium (Welgene) supplemented with 10% FBS and penicillin/streptomycin (100 U/100 μg).

### Induction of cell death

Apoptosis was induced by exposing epithelial cancer cell lines to UV irradiation at 254 nm for 15 min followed by incubation in RPMI 1640 medium supplemented with 10% FBS for 2 h at 37 °C in 5% CO_2_. Necrotic (i.e., lysed) cancer cells were obtained by multiple freeze‒thaw cycles. Apoptosis and necrosis were confirmed by annexin V-FITC/propidium iodide (BD Biosciences, San Jose, CA, USA) staining followed by flow cytometric analysis on a FACSCalibur system (ACEA Novocyte 3000, Agilent, Santa Clara, CA, USA) [18]. Supplementary Fig. S1a, b show representative dot plots indicating the percentages of apoptotic and necrotic 344SQ and A549 cells.

### Incubation of CAFs and preparation of CAF CM

CAFs were plated at 3 × 10^5^ cells/ml and grown in suitable medium at 37 °C in 5% CO_2_. After overnight incubation, the cells were serum-starved with X-VIVO 10 medium (04-380Q, Lonza, Basel, Switzerland) for 24 h before stimulation. For stimulation, the culture medium was replaced with X-VIVO 10 medium containing apoptotic or necrotic cancer cells (9 × 10^5^ cells/ml). After 20 h, the supernatant was harvested by centrifugation and used as the CM for the stimulation of epithelial cancer cells (5 × 10^5^ cells/ml) or CAFs (3 × 10^5^ cells/ml).

### Migration and invasion assays

Cell migration and invasion were assessed using Transwell chambers (Corning Inc., Corning, NY, USA) coated with 30 μg/ml fibronectin and 300 μg/ml Matrigel matrix, respectively, according to the manufacturer’s instructions. Briefly, cancer cells (1 × 10^5^ cells/well) preincubated in CM from CAFs in the absence or presence of TGF-β1 (10 ng/ml) for 48 h were plated in replicate wells with serum-free RPMI in the upper chamber and RPMI 1640 medium supplemented with 10% FBS in the bottom chamber at 37 °C for 16 h (migration assay) or 24 h (invasion assay). Migration and invasion assays were also performed with CAFs after direct exposure to ApoSQ stimulated with TGF-β1 (10 ng/ml) for 24 h. Before TGF-β1 stimulation, CAFs were washed with X-VIVO 10 medium after direct exposure to apoptotic or necrotic cancer cells. After fixation with 4% paraformaldehyde, nonmigrated or noninvaded cells on the upper surface of the membrane were removed by scraping with a cotton swab. Cells on the lower surface were stained with 0.1% crystal violet and washed with distilled water. Cells in three random microscopic fields (×10 magnification) were photographed and counted.

### Immunoblot analysis

Standard western blotting was performed using whole-cell extracts. Antibody information is provided in Table S1. Whole-cell extracts were prepared from CAFs or cancer cells exposed to apoptotic cells or CM. Cells were harvested, washed with ice-cold phosphate-buffered saline (PBS), and lysed in radioimmunoprecipitation assay buffer [10 mM Tris (pH 7.2), 150 mM NaCl, 1% Nonidet P-40, 0.5% sodium deoxycholate, 0.1% SDS, 1.0% Triton X-100, and 5 mM EDTA] supplemented with protease inhibitors for 30 min on ice. Equal amounts of protein were resolved on SDS‒PAGE gels (#161-0158, Bio-Rad Laboratories, Hercules, CA, USA) and transferred to nitrocellulose membranes (10600001, GE Healthcare Life Science, Piscataway, NJ, USA) using a wet transfer system (Bio-Rad Laboratories). After blocking with 5% bovine serum albumin (BSA)-TBST or 5% milk-TBST for 1 h, the membranes were incubated first with the indicated primary antibodies overnight and then with the indicated secondary antibody for 1 h at 37 °C. An Odyssey image analysis system (LI-COR Biosciences, Lincoln, NE, USA) was used for quantification. Antibody information is provided in Table S1.

### Quantitative real-time PCR

Total RNA was extracted from isolated cells using TRIzol reagent (RNAiso plus, Takara Bio Inc., Kusatsu, Japan). After reverse transcription with a ReverTra Ace^™^ qPCR RT Kit and Master Mix (Toyobo, Osaka, Japan), SYBR Green-based quantitative real-time polymerase chain reaction (qRT‒PCR) was performed using a QuantStudio^™^ 3 Real-Time PCR System (Applied Biosystems, Foster City, CA, USA). mRNA levels were normalized to *Hprt* mRNA levels and are reported as fold changes in expression compared with the control group. The primer sequences used to amplify the target genes are listed in Table S2.

### Cytokine array analysis

Cytokine array analysis was performed with CAF CM, ApoSQ-CAF CM, and ApoSQ CM using a Proteome Profiler Mouse XL Cytokine Array Kit (#ARY028, R&D Systems, USA) following the manufacturer’s instructions.

### Immunofluorescence

CAFs grown on glass coverslips until confluent were fixed with 4% paraformaldehyde for 8 min at room temperature. For staining of paraffin-embedded tumor samples, formalin fixation was performed at room temperature for 30 min, and IF-Wash buffer (0.05% NaN3, 0.1% BSA, 0.2% Triton X-100, and 0.05% Tween 20 in PBS) was used. After fixation, samples were washed three times with wash buffer for 5 min each and permeabilized with 0.5% Triton X-100 (Sigma‒Aldrich) in PBS at RT for 5 min. BSA (5%) in PBS with and without mouse IgG blocking reagent was used for immunohistochemistry and immunocytochemistry, respectively. After 1 h, target proteins were captured by each primary antibody during incubation for 18 h at 4 °C. Captured proteins were detected and visualized by incubation with fluorescently labeled IgG in a dark room for 1 h. After staining, slides were mounted with VECTASHIELD mounting medium containing DAPI (Vector Laboratories, Burlingame, CA, USA) and imaged with a confocal microscope (LSM5 PASCAL, Carl Zeiss, Jena, Germany) or an inverted fluorescence microscope (Eclipse Ti2-U, Nikon, Tokyo, Japan). Information on the antibody sources and dilution ratios is provided in Table S1.

### ELISA

WISP-1 and LIF in CM and serum were measured using ELISA kits (R&D Systems) following the manufacturer’s instructions.

### Transient transfection and luciferase activity assay

CAFs were transiently transfected with siRNA specifically targeting WISP1 (Bioneer Inc, Daejeon, Korea), LIF (Bioneer), Notch1 (Bioneer), or BAI1 (Dharmacon. CO, USA) or control siRNA (Bioneer, Dharmacon) at a final concentration of 100 nM using a transfection reagent (Lipofectamine RNAi MAX; Invitrogen, Carlsbad, CA, USA) according to the manufacturer’s instructions. After overnight transfection, the cells were cultured in suitable medium for 24 h and stimulated with ApoSQ. 344SQ cells were transiently transfected with Dll1-specific siRNA (D-050912-01-0020, Dharmacon) or control siRNA before exposure to UV irradiation. The siRNA sequences used for targeting genes were as follows (gene: sense, antisense). WISP-1: 5′-GGAAUCCUAACGAUAUCUU-3′, 5′- AAGAUAUCGUUAGGAUUCC-3′; LIF: 5′-CGACCACUCUGACAAAGAA-3′, 5′- UUCUUUGUCAGAGUGGUCG-3′; Notch1: 5′-CCCUUUGAGUCUUCAUACA-3′, 5′- UGUAUGAAGACUCAAAGGG-3′; Dll1: 5′-GCACGGACCUUGAGGACAG-3′, 5′-CUGUCCUCAAGGUCCGUGC-3′; BAI1: 5′-GUGUCAUCCUCGUACAGU-3′, 5′- ACUGUACGAGGAUGACAC3′.

Gene overexpression experiments were performed with CAFs seeded at 0.5 × 10^6^ cells/ml in 60 mm dishes for 24 h. For WISP-1 overexpression, cells were transfected with 2.5 μg of the mouse WISP-1 gene ORF cRNA clone expression plasmid (MG5A0255-CH, Sino Biological, North Wales, PA, USA) using Lipofectamine 2000 (Thermo Fisher Scientific, Waltham, MA, USA) for 24 h according to the manufacturer’s instructions. Mock-transfected cells were transfected with a control vector, pcDNA3, by the same method used for WISP-1 transfection. For BAI1 overexpression, CAFs were transfected with 2.0 μg of the empty pEBB vector (mock) or pEBB-BAI-Flag plasmid (a gift from Dr. Daeho Park, Gwangju Institute of Science and Technology, Gwangju, Korea) with Lipofectamine 2000 reagent (Thermo Fisher Scientific) for 48 h according to the manufacturer’s instructions. WISP-1 and BAI1 gene and protein expression were measured in cell lysates to confirm plasmid efficiency.

For luciferase assays, CAFs were transfected using Lipofectamine LTX transfection reagent and PLUS reagent (Life Technologies, Darmstadt, Germany). Cells were transfected with 800 ng/well 4× CSL luciferase plasmid (#41726, Addgene, Watertown, MA, USA), which produces luciferase in response to Notch pathway activation, and 200 ng/well Renilla luciferase plasmid. Firefly luciferase activity was normalized to Renilla luciferase activity, and values are reported as fold changes relative to the values in cells transfected with the PGL2 control vector. All conditions were analyzed in triplicate in each independent experiment. Luciferase assays were performed using the Dual-Luciferase Assay System (Promega, Madison, WI, USA).

### WISP-1 neutralization in CM

CM from CAFs was incubated for 2 h with 10 μg/ml mouse anti-mouse WISP-1 neutralizing antibody (R&D Systems) or 10 μg/ml IgG isotype control (R&D Systems). The neutralization efficiency of the anti-WISP-1 antibody was tested by WISP1 ELISA before use.

### Flow cytometry

For Notch ligand staining, UV-irradiated apoptotic cancer cells and viable cancer cells were incubated with an anti-Dll1, anti-Dll3, anti-Dll4, anti-Jag1, or anti-Jag2 antibody (1:100) in FACS buffer (PBS with 0.1% BSA and 0.1% sodium azide) for 30 min at RT. The cells were then incubated with an Alexa 488- or 594-conjugated secondary antibody in FACS buffer for 30 min at RT. After incubation, the cells were washed three times with FACS buffer, and the expression of Notch ligands was analyzed by flow cytometry (ACEA Novocyte 3000, Agilent, Santa Clara, CA, USA).

### Phagocytosis assays

Phagocytosis of apoptotic cancer cells was assessed using flow cytometric and immunofluorescence analyses as described previously [20]. Briefly, CAFs were stained with PKH26 (red) before coculture with PKH67 (green)-labeled apoptotic 344SQ cells at a 1:3 ratio for 24 h, and the phagocytosis rate was evaluated using two-color flow cytometry. For immunofluorescence analysis, PKH67-labeled apoptotic 344SQ cells were cocultured with CAFs for 24 h. After washing, the CAFs were fixed with 3.7% w/v paraformaldehyde and treated with 0.1% Triton X-100 for 15 min for permeabilization. F-actin was stained with rhodamine phalloidin (Invitrogen) according to the manufacturer’s instructions. Images were acquired with a confocal microscope (LSM5 PASCAL; Carl Zeiss, Jena, Germany). The phagocytic index was calculated as (number of apoptotic bodies)/(200 CAFs) × 100.

### Rac1 activity assay

CAFs were preincubated with DAPT (10 or 20 µM) for 2 h and transfected with Notch1 or control siRNA for 24 h before treatment with ApoSQ (1:3 ratio) for 24 h. Rac1 activity was assessed using a G-LISA Rac1 activation assay kit (Cytoskeleton, Denver, CO, USA) according to the manufacturer’s instructions. Briefly, cell lysates containing the same quantity of protein were added to Rac1-GTP binding plates and incubated at 4 °C for 30 min. The plates were washed with PBS containing 0.05% Tween 20 and then incubated with an antigen-presenting buffer. After washing, the plates were incubated with an anti-Rac1 primary antibody and a secondary antibody. Immunoreactions were detected with horseradish peroxidase detection reagents and terminated with stop solution. The plates were read with a microplate spectrophotometer to measure the absorbance at 490 nm.

### Mouse experiments

The Animal Care Committee of the Ewha Medical Research Institute approved the experimental protocol (EUM19-430). Mice were cared for and handled in accordance with the National Institutes of Health Guide for the Care and Use of Laboratory Animals. Lung cancer metastasis assays with syngeneic tumors were performed as previously described [18, 21]. Briefly, 344SQ cells (1 × 10^6^ cells in 100 μl of PBS per mouse) were subcutaneously injected into the right posterior flanks of syngeneic (129/Sv) mice (male, age 8 weeks, *n* = 9–12 per group). Two days after the first injection, a second injection of 100 μl of PBS with or without 1 × 10^7^ apoptotic 344SQ cells was performed in the same lesion. For the Notch1 inhibition experiments, the selective Notch1 inhibitor LY3039478 (8 mg/kg) was administered orally in 15% sugar gel vehicle three times a week for 6 weeks [22] beginning the day before injection of ApoSQ. For the CM experiments, CM (100 μl per mouse) derived from CAFs treated with the neutralizing anti-WISP-1 antibody (10 μg/ml) or IgG isotype control was administered via intratumoral injection three times a week starting 2 days after 344SQ cell injection (*n* = 8 mice per group). In addition, rWISP-1 (12.5 and 25 μg/kg) [23] was administered via intratumoral injection three times a week starting 2 days after 344SQ cell injection (*n* = 8 mice per group). Mice were monitored daily for tumor growth and sacrificed 6 weeks after injection. Necropsies were performed to examine the diameter and weight of the subcutaneous tumor masses; lung metastasis status (i.e., number of nodules or incidence); and histological characteristics of formalin-fixed, paraffin-embedded, immunofluorescence-stained primary tumors. All animal testing and research were performed with age-matched male mice.

### Isolation of Thy1^+^ CAFs, CD326^+^ tumor cells, and CD11b^+^ tumor-associated macrophages from primary tumors

Isolation of single cells from mouse tumors was performed following a previously described process with modifications [24]. Necropsied fresh tumors were disaggregated and dissociated with a Tumor Dissociation Kit (Miltenyi Biotec, Auburn, CA, USA) according to the manufacturer’s instructions. Cell suspensions were filtered through 70- and 40 μm sterile nylon meshes and incubated with red blood cell lysis buffer. After centrifugation at 300 × *g* for 10 min, the remaining cells were labeled with anti-CD45 (Abcam, Cambridge, UK), anti-CD68 (Abcam), anti-CD31 (Abcam), and anti-CD326 (BD Bioscience, San Diego, CA, USA) antibodies for selection of non-CAFs. Labeled cells were depleted from the whole-cell suspension with prewashed Dynabeads^TM^ (Invitrogen), and cells were separated from the magnetic beads using a PureProteome^™^ Magnetic Stand (Millipore, Billerica, MA, USA). Thy1^+^ CAFs in the supernatant of unlabeled cells were sorted using CD90.2 MicroBeads and a MACS MS column (Miltenyi Biotec) according to the manufacturer’s instructions. CD326^+^ epithelial tumor cells and CD11b^+^ tumor-associated macrophages (TAMs) in the supernatant of negatively selected cells, were isolated with CD326 and CD11b MicroBeads (Miltenyi Biotec), respectively. Isolated cells were cultured with complete media as follows: α-MEM (Welgene) containing 20% FBS and 1% penicillin‒streptomycin for Thy1^+^ cells; RPMI 1640 medium containing 10% FBS and 1% penicillin‒streptomycin for CD326^+^ cells; and DMEM (Welgene) containing 10% FBS and 1% penicillin‒streptomycin for CD11b^+^ cells. Groups of isolated individual cells were verified using qRT‒PCR. Individual cells were isolated from two or three randomly selected mouse primary tumors in each group.

### Reverse transcription PCR array assay

To profile the expression of genes associated with tumor metastasis in isolated CD326^+^ tumor cells and with extracellular matrix (ECM) and adhesion molecules in Thy1^+^ CAFs isolated from primary tumors, we employed a Mouse Tumor Metastasis RT^2^ Profiler^™^ PCR Array (PAMM-028ZA-6, Qiagen, Hilden, Germany) and Mouse Extracellular Matrix and Adhesion Molecules RT^2^ Profiler^™^ PCR Array (PAMM-013ZA-6, Qiagen). RNA isolation, DNase treatment, and RNA cleanup were performed according to the manufacturer’s instructions (Takara Bio). Isolated RNA was reverse-transcribed into cDNA using an RT^2^ First Strand Kit (Qiagen). PCR was performed using RT^2^ SYBR Green qPCR Master Mix (Qiagen) on a QuantStudio^™^3 Real-Time PCR System and an ABI Prism 7900 instrument (Applied Biosystems). Expression data were normalized to the average Ct values of glyceraldehyde 3-phosphate dehydrogenase *(Gapdh*), the housekeeping gene in the array. Each assay was conducted in triplicate.

### Statistical analysis

Pairwise comparisons were performed using two-tailed Student’s *t* test, and multiple comparisons were performed using the Kruskal‒Wallis test followed by Dunn’s post hoc test. *P* values < 0.05 were considered statistically significant. All data were analyzed using Prism 5 software (GraphPad Software Inc., San Diego, CA, USA).

## Results

### CM from CAFs exposed to apoptotic cancer cells inhibits cancer cell migration and invasion

**Fig. 1:**
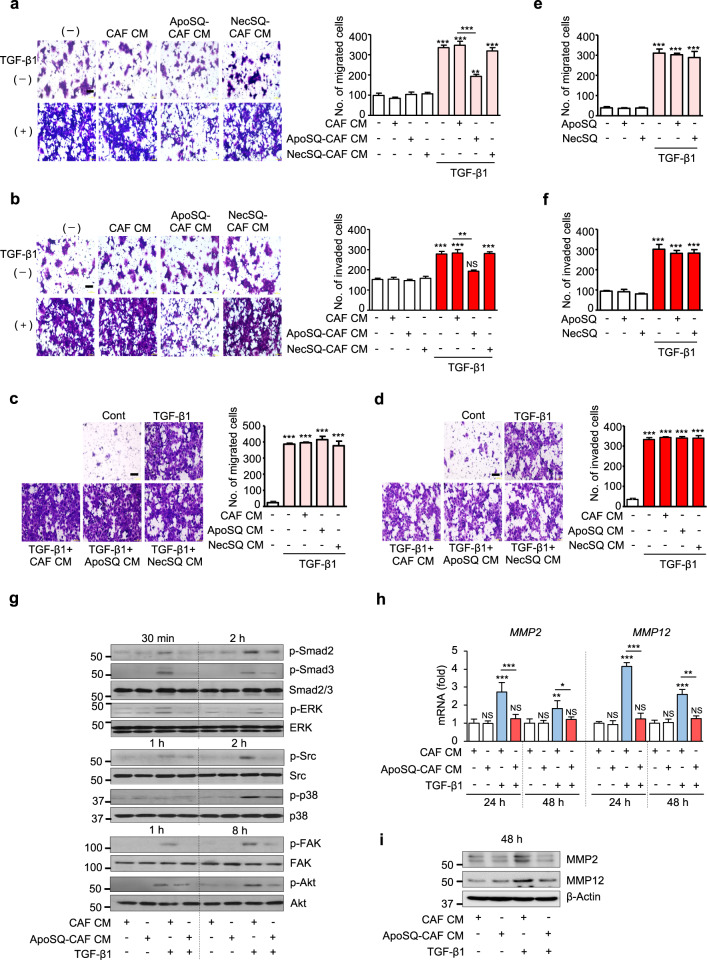
CM from CAFs exposed to apoptotic lung cancer cells inhibits TGF-β1 signaling pathways and the migration and invasion of lung cancer cells. **a**, **c** Phase contrast microscopy (*left*) and quantification of migrated 344SQ cells (*right)* using Fn-coated Transwell plates. **b**, **d** Phase contrast microscopy (*left*) and quantification of invaded 344SQ cells (*right)* using Matrigel-coated Transwell plates. Scale bars: 100 µm. Quantification of migrated (**e**) and invaded (**f**) 344SQ cells. **g** Immunoblot analysis of the indicated proteins in 344SQ cell lysates. **h** qRT‒PCR of MMP2 and MMP12 in 344SQ samples. **i** Immunoblot analysis of MMP2 and MMP12 in 344SQ cell lysates. **a**, **b**, **g**–**i** CAFs were exposed to ApoSQ or NecSQ for 20 h. CAF CM, ApoSQ-CAF CM, or NecSQ-CAF CM was added to 344SQ cells with or without TGF-β1 (10 ng/ml) for 48 h or the indicated duration. **c**, **d** ApoSQ CM or NecSQ CM was added to 344SQ cells with TGF-β1 (10 ng/ml) for 48 h. **e**, **f** ApoSQ or NecSQ were directly added to 344SQ cells with or without TGF-β1 (10 ng/ml) for 48 h. NS: not significant, *P < 0.05, **P < 0.01, ***P < 0.001, two-tailed Student’s *t* test. The data are from one experiment representative of three independent experiments with similar resul*t*s (**a**–**d** *left*, **g**, **i**) or from three independent experiments (mean ± standard error in **a**–**d** *right*, **e**, **f**, **h**)

Paracrine communication between CAFs and cancer cells facilitates cancer cell migration and invasion [5]. Thus, we first examined whether exposure of CAFs to UV-irradiated apoptotic cancer cells inhibits lung cancer cell migration and invasion via secretion of bioactive mediators. CAFs were isolated from lung tumors of Kras-mutant (Kras^LA1^) mice [19, 25] using Thy1, a fibroblast-specific marker [24]. Here, 344SQ cells were mainly utilized for in vitro and in vivo studies because 344SQ is a highly invasive and metastatic lung adenocarcinoma cell line derived from mice that coexpress the Kras^LA1^ and 53^R172H^ alleles and has been demonstrated to form lung metastases when reimplanted syngeneically into wild-type mice [26]. CAFS were treated for 20 h with ApoSQ. 344SQ cells were treated with CM from CAFs exposed to ApoSQ (ApoSQ-CAF CM) or necrotic cells (NecSQ-CAF CM) in the presence or absence of TGF-β1 for 48 h. Transwell migration and invasion assays were performed to assess cell motility and invasiveness toward a chemoattractant gradient. We found that ApoSQ-CAF CM prevented TGF-β1-induced migration and invasion of cancer cells, whereas control (CAF CM) or NecSQ-CAF CM did not (Fig. 1a, b). We confirmed the antimigratory and anti-invasive effects of CM from CAFs exposed to different types of apoptotic cancer cells with the human non-small cell lung cancer A549 (Supplementary Fig. S2a, b) and colon cancer HCT116 (Supplementary Fig. S2c, d) cell lines. Notably, neither CM from nor direct exposure to ApoSQ or NecSQ alone influenced the migration or invasion of 344SQ cells (Fig. 1c–f and Supplementary Fig. S2e, f).

Activation of TGF-β signaling induces cancer cell migration and invasion by regulating Smad-dependent and Smad-independent pathways [27, 28]. These pathways modulate the activity of matrix metalloproteinases (MMPs) in both cancer cells and tumor stroma-associated cells. In particular, MMP2 and MMP12 have been shown to degrade components of the ECM and correlate with lung cancer metastasis [29, 30]. In the present study, we found that ApoSQ-CAF CM blocked TGF-β1 signaling, including signaling through the Smad2/3, ERK, Src, p38 MAP kinase, FAK, and Akt pathways, in 344SQ cells (Fig. 1g). ApoSQ-CAF CM also reversed the TGF-β1 signaling-induced upregulation of MMP-2 and MMP-12 expression at the mRNA and protein levels (Fig. 1h, i). Collectively, these data suggest that CM from CAFs exposed to apoptotic cancer cells inhibits the migration and invasion of lung cancer cells via inhibition of TGF-β1 signaling-related pathways as well as MMP-2 and MMP-12 expression.

### Interaction of CAFs with apoptotic lung cancer cells inhibits CAF migration and invasion

**Fig. 2:**
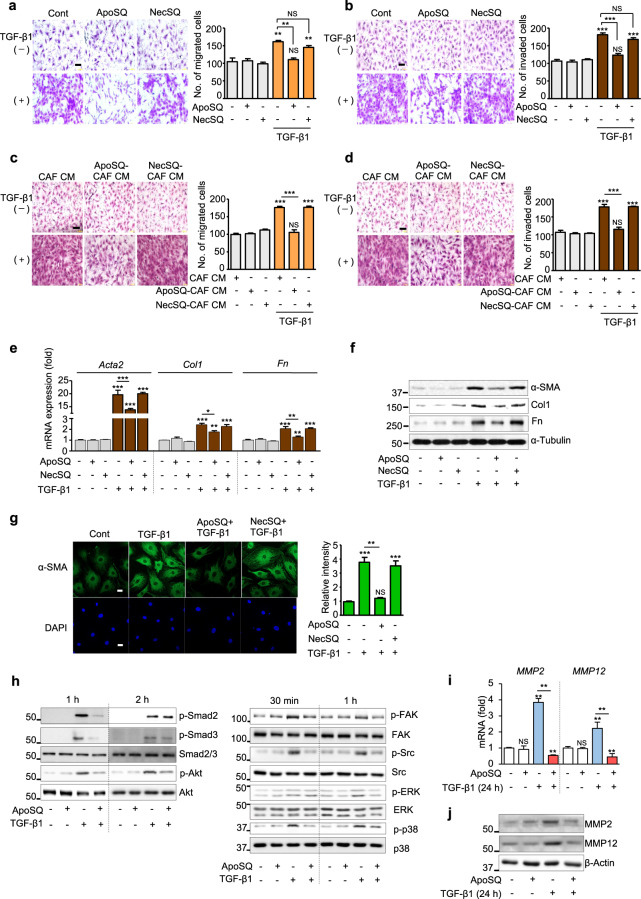
Interaction between CAFs and apoptotic lung cancer cells inhibits TGF-β1 signaling, migration and invasion and the expression of activation markers in CAFs. Phase contrast microscopy (*left*) and quantification of migrated (**a**, **c**) and invaded (**b**, **d**) CAFs (*right)*. Scale bars: 100 µm. **a**, **b** CAFs were exposed to ApoSQ or NecSQ for 20 h, which the medium was then replaced with fresh medium with or without TGF-β1 (10 ng/ml) for 24 h. **c**, **d** CAFs were exposed to ApoSQ or NecSQ for 20 h. CAF CM, ApoSQ-CAF CM, or NecSQ-CAF CM was added to naïve CAFs with or without TGF-β1 (10 ng/ml) for 24 h. **e** qRT‒PCR analysis of CAF activation markers in CAF lysates. **f** Immunoblot analysis of the indicated proteins in CAF lysates. **g** Immunofluorescence staining (green; *left*) and quantification (*right*) of α-SMA in CAFs. Original magnification: ×400. Scale bars = 20 μm. The imaging medium was VECTASHIELD fluorescent mounting medium containing DAPI. **h**, **j** Immunoblot analysis of the indicated proteins in CAF lysates. **i** qRT‒PCR of MMP2 and MMP12 in CAF samples. **e**–**j** CAFs were exposed to ApoSQ or NecSQ for 20 h, which the medium was then replaced with fresh medium with or without TGF-β1 (10 ng/ml) for 24 h or the indicated duration. NS: not significant, *P < 0.05, **P < 0.01, ***P < 0.001, two-tailed Student’s *t* test. The data are from one experiment representative of three independent experiments with similar results (**a**–**d** and **g** *left*, **f**, **h**, **j**) or from three independent experiments (mean ± standard error; **a**–**d** and **g** *right*, **e**, **i**)

The migratory ability of CAFs is dependent on their activation status. We found that direct exposure of CAFs to ApoSQ inhibited TGF-β1-induced migration and invasion, whereas exposure to NecSQ had no inhibitory effects (Fig. 2a, b). Similar to the direct effects of ApoSQ, ApoSQ-CAF CM inhibited TGF-β1-induced migration and invasion of CAFs, whereas NecSQ-CAF CM had no inhibitory effects (Fig. 2c, d). CM from CAFs exposed to other types of apoptotic cancer cells, such as A549 cells (Supplementary Fig. S3a, b) and HCT116 cells (Supplementary Fig. S3c, d), also had antimigratory and anti-invasive effects. Notably, CM from ApoSQ or NecSQ alone did not have antimigratory or anti-invasive effects (Supplementary Fig. S3e).

ApoSQ but not NecSQ suppressed TGF-β1-induced mRNA and protein expression of the CAF activation markers α-smooth muscle actin (SMA), collagen type 1, and fibronectin (Fig. 2e, f). The most widely used marker for CAFs is α-SMA because this protein is a specific marker for myofibroblasts [31]. Thus, α-SMA protein expression was analyzed by immunocytochemistry to evaluate the CAF activation level. The results of immunocytochemical analysis confirmed the reduction in α-SMA protein expression in CAFs by exposure to ApoSQ but not NecSQ (Fig. 2g). Similarly, ApoSQ inhibited TGF-β1-induced activation of Smad and non-Smad signaling pathways in CAFs (Fig. 2h). ApoSQ also reduced TGF-β1-induced MMP-2 and MMP-12 expression at the mRNA and protein levels (Fig. 2I, j). Collectively, these data indicate that the interaction of CAFs with apoptotic lung cancer cells inhibits CAF migration and invasion via inhibition of TGF-β1 signaling pathways as well as MMP-2 and MMP-12 expression.

### WISP-1 secretion is required for the observed antimigratory and anti-invasive effects

**Fig. 3:**
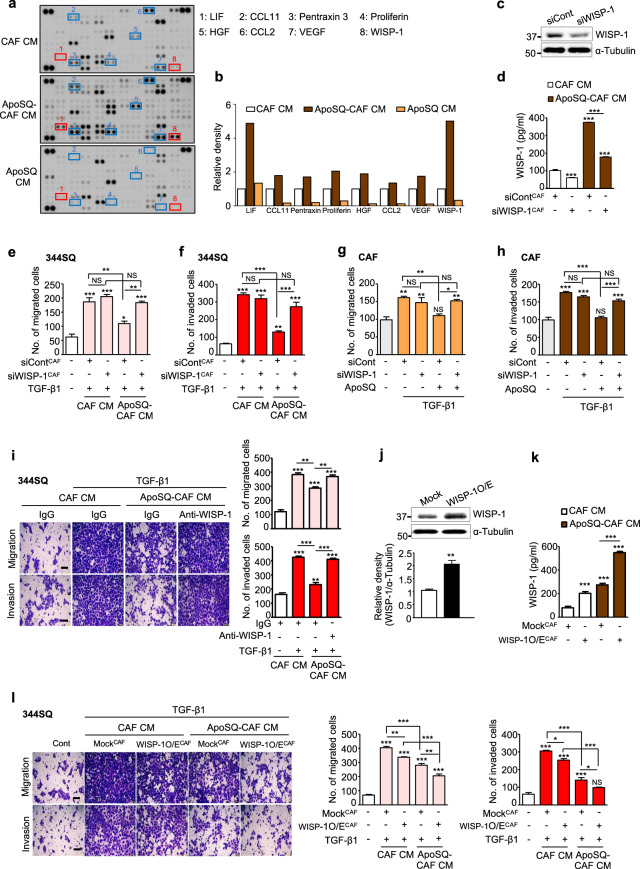
WISP-1 is required for the antimigratory and anti-invasive effects. **a** Cytokine array analysis of CAF CM, ApoSQ-CAF CM, or ApoSQ CM for 24 h. **b** Relative image densities of cytokine spots for ApoSQ-CAF CM and ApoSQ CM compared with CAF CM. **c** Immunoblot analysis of WISP-1 in CAFs transfected with WISP-1 siRNA (*upper*). Densitometric analysis of the relative WISP-1 abundance (*lower*). **d** ELISA of WISP-1 in CM from CAFs transfected with WISP-1 siRNA for 24 h before treatment with or without ApoSQ for 20 h. Quantification of migrated (**e**) and invaded (**f**) 344SQ cells. **e**, **f** CAFs were transfected with control or WISP-1 siRNA before exposure to ApoSQ for 20 h. CM was added to 344SQ cells with TGF-β1 (10 ng/ml) for 48 h. Quantification of migrated (**g**) and invaded (**h**) CAFs. **g**, **h** CAFs were transfected with WISP-1 siRNA before exposure to ApoSQ for 20 h, which the medium was then replaced with fresh medium containing TGF-β1 (10 ng/ml) for 24 h. **i** Phase contrast microscopy (*left*) and quantification of migrated and invaded 344SQ cells (*right)*. CAF CM and ApoSQ-CAF CM were treated with a neutralizing anti-WISP-1 Ab (10 μg/ml) or IgG isotype control before addition to 344SQ cells with or without TGF-β1 (10 ng/ml) for 48 h. **j** Immunoblot analysis of WISP-1 in CAFs transfected with control vector (mock) or the WISP-1 plasmid (WISP-1 O/E) for 24 h (*upper*). Densitometric analysis of the relative WISP-1 protein abundance (*lower*). **k** ELISA of WISP-1 in CM from CAFs transfected with empty vector or the WISP-1 plasmid for 24 h before treatment with or without ApoSQ for 20 h. **l** Phase contrast microscopy (*left*) and quantification of migrated and invaded 344SQ cells (*right*). CM from CAFs transfected with empty vector or the WISP-1 plasmid before treatment with or without ApoSQ for 20 h was added to 344SQ cells with TGF-β1 (10 ng/ml) for 48 h. **i**, **l** Scale bars: 100 μm. NS, NS: not significant, *P < 0.05, **P < 0.01, ***P < 0.001, two-tailed Student’s *t* test. The data are from one experiment representative of three independent experiments with similar results (**c** and **j** *upper*, **i** and **l** *left*) or from three independent experiments (mean ± standard error; **c** and **j** *lower*, **d**–**h**, **i** and **l** *right*, **k**)

To determine whether paracrine/autocrine factors secreted from CAFs are critical for inhibiting migration and invasion, we performed cytokine array analysis to determine the levels of up to 111 cytokines in a single sample. The levels of eight cytokines—WISP-1, leukemia inhibitory factor (LIF), proliferin, hepatocyte growth factor, chemokine ligand 11 (CCL11), vascular endothelial growth factor (VEGF), pentraxin 3, and CCL2—were increased in ApoSQ-CAF CM compared with CAF CM and ApoSQ CM. Among these cytokines, WISP-1 and LIF were the most highly increased (Fig. 3a, b). On the other hand, the levels of other cytokines, including interleukin-1 receptor antagonist (IL-1ra), CD14, coagulation factor III, and osteopontin, were increased in ApoSQ-CAF CM compared with CAF CM but were comparable to or less than those in ApoSQ CM (Supplementary Fig. S4a, b). Thus, these cytokines were also excluded from further study. Ultimately, we determined which cytokine inhibits migration and invasion using siRNAs specific for WISP-1 and LIF. WISP-1 knockdown reduced WISP-1 secretion from CAFs (Fig. 3c, d) and reversed the antimigratory and anti-invasive effects of ApoSQ-CAF CM and ApoSQ on 344SQ cells (Fig. 3e, f and Supplementary Fig. S5a) and CAFs (Fig. 3g, h and Supplementary Fig. S5b). In contrast, LIF knockdown, which reduced LIF secretion but not WISP-1 secretion from CAFs induced by ApoSQ (Supplementary Fig. S6a–c), did not affect the migration and invasion of 344SQ cells or CAFs (Supplementary Fig. S6d, e).

In addition, treatment with ApoSQ-CAF CM after neutralization with an anti-WISP-1 antibody reversed the antimigratory and anti-invasive effects on 344SQ cells, whereas treatment with control IgG had no effect (Fig. 3i). Notably, WISP-1 overexpression in CAFs enhanced WISP-1 secretion with or without exposure to ApoSQ (Fig. 3j, k). CM from WISP-1-overexpressing CAFs cultured with ApoSQ further inhibited the migration and invasion of 344SQ cells compared with CM from mock-transfected CAFs cultured with ApoSQ (Fig. 3l). Exposure of WISP-1-overexpressing CAFs to ApoSQ did not result in further inhibition, as the migration and invasion of CAFs were already at the basal levels observed after exposure of mock-transfected CAFs to ApoSQ (Supplementary Fig. S7). Collectively, these results suggest that WISP-1 secretion from CAFs exposed to ApoSQ mediates the antimigratory and anti-invasive effects on lung cancer cells and CAFs.

### WISP-1 signals via integrin αvβ3 in 344SQ cells and αvβ5 in CAFs to inhibit TGF-β1-induced migration and invasion

To validate the observation that WISP-1 acts in autocrine and paracrine manners to exert antimigratory and anti-invasive effects, 344SQ cells and CAFs were directly treated with rWISP-1. As expected, rWISP-1 inhibited the migration and invasion of 344SQ cells and CAFs in a dose-dependent manner (Supplementary Fig. S8a, b). rWISP-1 also blocked TGF-β1-induced Smad2/3 and non-Smad signaling pathway activation as well as MMP2/MMP12 mRNA and protein expression in 344SQ cells (Supplementary Fig. S8c–e) and CAFs (Supplementary Fig. S8f–h).

**Fig. 4:**
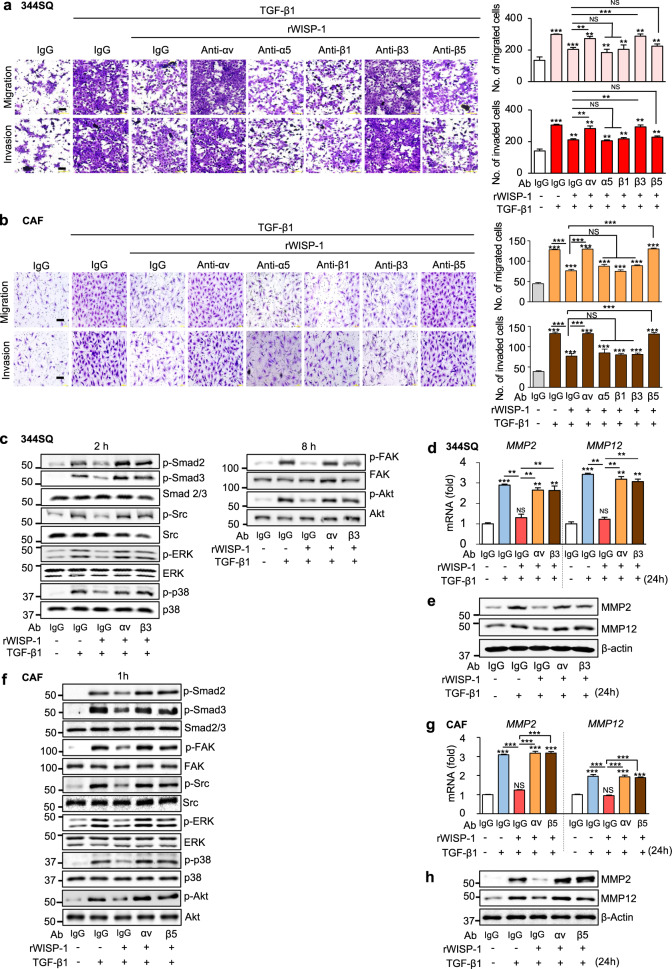
WISP-1 acts through integrin αvβ3 in 344SQ cells and αvβ5 in CAFs to inhibit TGF-β1-induced migration and invasion. **a** Phase contrast microscopy (*left*) and quantification of migrated and invaded 344SQ cells (*right)*. 344SQ cells were pretreated with an anti-integrin blocking antibody (3 μg/ml; anti-integrin αν, α5, β1, β3, or β5) or corresponding IgG isotype control for 30 min before treatment with rWISP-1 (10 ng/ml) and TGF-β1 (10 ng/ml) for 24 h. **b** Phase contrast microscopy (*left*) and quantification of migrated and invaded CAFs (*right)*. CAFs were pretreated with an anti-integrin blocking antibody (10 μg/ml) for 1 h before treatment with rWISP-1 (3 ng/ml) and TGF-β1 (10 ng/ml) for 24 h. Scale bars: 100 μm. Immunoblot analysis of the indicated proteins in 344SQ cell (**c**, **e**) and CAF lysates (**f**, **h**). qRT‒PCR analysis of *MM2* and *MMP12* mRNA expression in 344SQ cell (**d**) and CAF (**g**) samples. **c–h** 344SQ cells and CAFs were pretreated with an anti-integrin blocking antibody (anti-integrin αν, β3, or β5) before direct exposure to rWISP-1 (10 ng/ml for 344SQ cells, 3 ng/ml for CAFs) with or without TGF-β1 (10 ng/ml) for the indicated time. NS: not significant, **P < 0.01, ***P < 0.001, two-tailed Student’s *t* test. The data are from one experiment representative of three independent experiments with similar results (**a**, **b** *left*, **c**, **e**, **f**, **h**) or from three independent experiments (mean ± standard error; **a**, **b** *right*, **d**, **g**)

A previous study showed that WISP-1 affects cellular functions by binding to integrin cell surface receptors [32, 33]. Integrins regulate a variety of cellular responses through various combinations of α and β subunits [34, 35]. However, the specific integrins mediating the paracrine and autocrine functions of WISP-1 in lung cancer cells and CAFs were unclear, although integrin αvβ3 and αvβ5 in lung cancer cells have been suggested to be involved in inhibition of cell invasion and metastasis [36, 37]. In the present study, to identify the specific integrins that mediate the function of WISP-1 in 344SQ cells and CAFs, we utilized blocking antibodies against integrin αv, α5, β1, β3, or β5. Pretreatment of 344SQ cells with anti-integrin αv or β3 antibodies substantially reversed the antimigratory and anti-invasive effects of rWISP-1 compared with control IgG (Fig. 4a). In CAFs, the inhibitory effects of rWISP-1 were not observed after treatment with anti-integrin αv or β5 antibodies compared with control IgG (Fig. 4b). Notably, pretreatment of 344SQ cells with anti-integrin α5, β1, or β5 antibodies and CAFs with anti-α5, β1, or β3 antibodies had only minor effects on cell migration and invasion. Next, we further examined whether WISP-1 inhibits TGF-β1-induced signaling pathways through integrin αv and β3 in 344SQ cells and through integrin αv and β5 in CAFs. As expected, pretreatment of 344SQ cells with anti-integrin αv or β3 antibodies also attenuated the inhibitory effects of rWISP-1 on TGF-β1-induced signaling pathways as well as on MMP2/MMP12 mRNA and protein expression (Fig. 4c–e). In CAFs, treatment with anti-integrin αv or β5 antibodies reversed these inhibitory effects of rWISP-1 (Fig. 4f–h). Collectively, these results confirm that WISP-1 plays a critical role in both autocrine and paracrine manners mainly via integrin αvβ3 receptors in 344SQ cells and integrin αvβ5 receptors in CAFs that further blocks TGF-β1-induced signaling pathways to modulate cell migration and invasion.

### Apoptotic cancer cells trigger Notch1 signaling for WISP-1 production

**Fig. 5:**
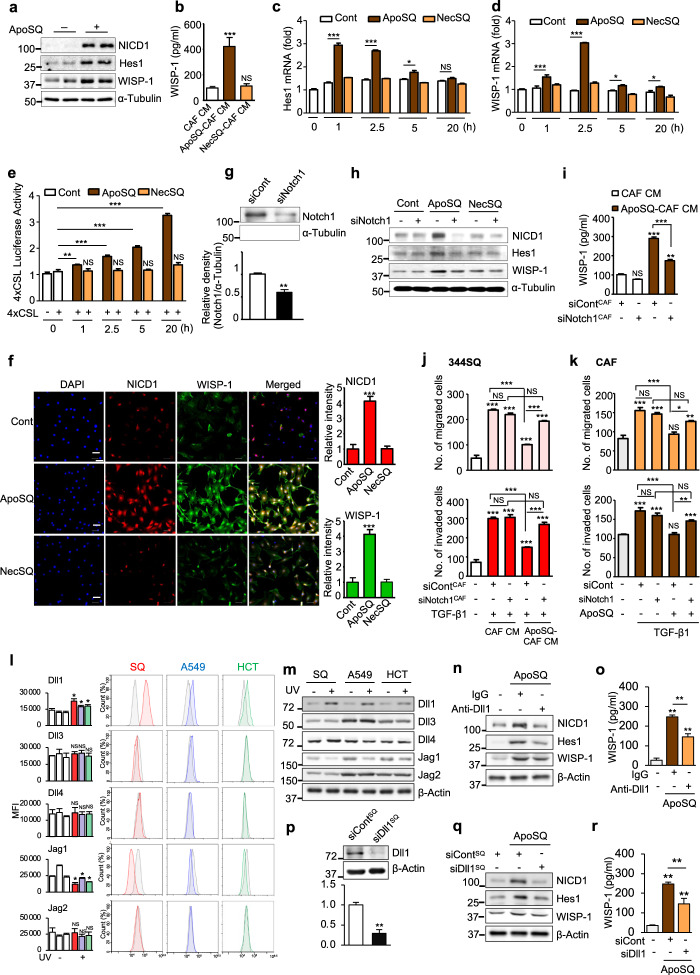
Apoptotic lung cancer cells trigger Notch1 signaling for WISP-1 production in CAFs via enhanced Dll1 expression. **a** Immunoblot analysis of proteins in CAFs stimulated with or without ApoSQ for 20 h. **b** ELISA of WISP-1 in CAF CM, ApoSQ-CAF CM, and NecSQ-CAF CM. **c**, **d** qRT‒PCR analysis of Hes1 and WISP-1 in CAFs at the indicated time points after stimulation with ApoSQ or NecSQ. **e** 4×CSL luciferase assay in CAFs at the indicated time points after stimulation with ApoSQ or NecSQ. **f** Immunofluorescence staining for NICD1 (red) and WISP-1 (green; *left*) and quantification (*right*) in CAFs stimulated with ApoSQ or NecSQ for 20 h. The imaging medium was VECTASHIELD fluorescent mounting medium containing DAPI. Original magnification: ×100. Scale bars = 100 μm. **g** Immunoblot analysis of Notch1 in CAFs transfected with control or Notch1 siRNA (*upper*). Densitometric analysis of the relative Notch1 abundance (*lower*). **h** Immunoblot analysis of the indicated proteins in CAFs transfected with Notch1 siRNA before stimulation with ApoSQ or NecSQ for 20 h. **i** ELISA of WISP-1 in CM from CAFs transfected with Notch1 siRNA before stimulation with or without ApoSQ for 20 h. Quantification of migrated and invaded 344SQ cells (**j**) and CAFs (**k**). **j** CAFs were transfected with control or Notch1 siRNA before exposure to ApoSQ for 20 h. CM was added to 344SQ cells with TGF-β1 (10 ng/ml) for 48 h. **k** CAFs were transfected with control or Notch1 siRNA before exposure to ApoSQ for 20 h, which the medium was then replaced with fresh medium containing TGF-β1 (10 ng/ml) for 24 h. **l** Flow cytometric analysis of Dll1, Dll3, Dll4, Jag1, and Jag2 expression in viable and UV-irradiated apoptotic cancer cells, including 344SQ, A549, and HCT116 cells. Mean fluorescence intensity (MFI) values (*left*) and histograms showing isotype control (gray) vs. specific Notch ligand antibody (red, blue, and green; *right*) staining. **m** Immunoblot analysis of Notch ligand protein expresison in viable and UV-irradiated apoptotic cancer cells. **n** Immunoblot analysis of proteins in CAFs exposed to ApoSQ for 20 h in the presence or absence of a neutralizing anti-Dll1 antibody (20 μg/ml). **o** ELISA of WISP-1 in CM from CAFs exposed to ApoSQ for 20 h in the presence or absence of a neutralizing anti-Dll1 antibody. **p** Immunoblot analysis of Dll1 in 344SQ cells transfected with Dll1 siRNA (*upper*). Densitometric analysis of the relative Dll1 abundance (*lower*). **q** Immunoblot analysis of proteins in CAFs exposed to ApoSQ. **r** ELISA of WISP-1 in CM from CAFs exposed to ApoSQ. **q**, **r** 344SQ cells were transfected with control or Dll1 siRNA before apoptosis induction by UV irradiation. CAFs were then stimulated with or without ApoSQ for 20 h. NS: not significant, *P < 0.05, **P < 0.01, ***P < 0.001, two-tailed Student’s *t* test. The data are from one experiment representative of three independent experiments with similar results (**a**, **f** *left*, **g** *upper*, **h**, **l** *right*, **m**, **n**, **p** *upper*, **q**) or from three independent experiments (mean ± standard error; **b-e**, **f** *right*, **g** *lower*, **i**–**k**, **l** *left*, **o**, **p** *lower*, **r**)

The Notch1-WISP-1 axis controls the regulatory role of stromal fibroblasts in melanoma invasion and metastasis [38]. Thus, we hypothesized that Notch1-WISP-1 signaling induced by ApoSQ plays a crucial role in the antimigratory and anti-invasive effects on lung cancer cells and CAFs. To test this hypothesis, we first examined whether Notch1 signaling in CAFs is activated by ApoSQ stimulation. Western blot analysis showed that ApoSQ enhanced the expression of Notch intracellular domain 1 (NICD1), which is only released upon activation of the Notch1 pathway [39], and of Hes1 and WISP-1, which are downstream targets of the Notch pathway [38]; in addition, it increased WISP-1 secretion (Fig. 5a, b). Hes1 and WISP-1 mRNA expression levels were also increased after exposure to ApoSQ, whereas NecSQ exposure had no such effects (Fig. 5c, d). Consistent with this finding, up to 20 h of exposure to ApoSQ increased the activity of the Notch-responsive reporter 4 × CSL-Luc, the universal transcriptional effector of Notch signaling, whereas no such activity was induced by NecSQ exposure (Fig. 5e). Furthermore, immunocytochemical analysis showed enhanced NICD1 and WISP-1 staining in CAFs exposed to ApoSQ (Fig. 5f), with WISP-1 staining colocalized with NICD1 staining in the nucleus.

Using Notch1 siRNA and a pharmacologic inhibitor, we further confirmed that Notch1-WISP-1 signaling induced by ApoSQ mediates the inhibited migration and invasion of 344SQ cells and CAFs. Notch1 knockdown reversed the increases in NICD1, Hes1, and WISP-1 protein expression and WISP-1 secretion induced by ApoSQ (Fig. 5g–i), and this effect was associated with suppression of the antimigratory and anti-invasive effects of ApoSQ-CAF CM on 344SQ cells (Fig. 5j and Supplementary Fig. S9a) and of exposure to ApoSQ on CAFs (Fig. 5k and Supplementary Fig. S9b). Similarly, treatment with 10 μM DAPT, a γ-secretase inhibitor, inhibited Notch1 activation and WISP-1 secretion induced by ApoSQ (Supplementary Fig. S9c, d) in CAFs and reversed the antimigratory and anti-invasive effects (Supplementary Fig. S9e, f). Collectively, these data indicate that apoptotic lung cancer cells trigger Notch1 signaling for WISP-1 production in CAFs, thereby preventing the migration and invasion of lung cancer cells and CAFs.

### Dll1 expression is enhanced in UV-irradiated apoptotic cancer cells

Most Notch signaling is initiated by interactions between the cell surface receptor Notch and a cell-bound ligand, such as Dll1, 3, or 4 or Jagged-like (Jag) 1 or 2 [40]. To investigate the cell surface interaction required for the activation of Notch1 signaling, we first examined the expression of Notch ligands on the surface of apoptotic cells using flow cytometry. Dll1 expression was enhanced in UV-irradiated apoptotic cancer cells, including 344SQ, A549, and HCT116 cells (Fig. 5l). However, the expression of other ligands either did not change or was decreased. Western blot confirmed that Dll1 expression was increased only in lysates of apoptotic cancer cells (Fig. 5m). Neutralization of Dll1 on the surface of ApoSQ with an anti-Dll1 antibody diminished Notch1 signaling in and WISP-1 secretion from CAFs induced by ApoSQ (Fig. 5n, o). Similarly, knockdown of Dll1 in 344SQ cells by transfection with a specific siRNA before apoptosis induction by UV irradiation inhibited Notch1 activation in and WISP-1 secretion from ApoSQ (Fig. 5p–r). These findings suggest that Notch1 signaling in CAFs is initiated by interaction with adjacent apoptotic cancer cells expressing Dll1.

### BAI1/Rac1 signaling facilitates efferocytosis by CAFs and participates in crosstalk with Notch1 signaling

**Fig. 6:**
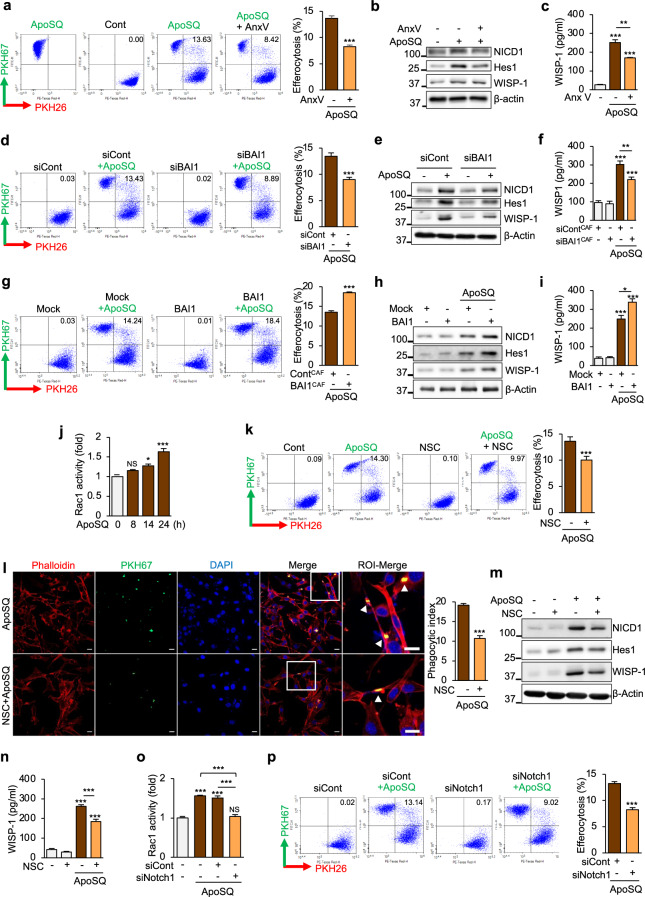
BAI1/Rac1 signaling facilitates efferocytosis and participates in crosstalk with Notch1 signaling in CAFs. Phagocytic activity of CAFs toward ApoSQ in the absence or presence of annexin V (AnxV, 10 μg/ml; **a**), transfected with BAI1 siRNA (**d**) or the BAI1-Flag construct (**g**), pretreated with a Rac1 inhibitor (NSC23766, 100 μM; **k**), or transfected with Notch1 siRNA (**p**). Representative dot plots showing the percentages of phagocytosing CAFs (PKH26^+^/PKH67^+^) and nonphagocytosing CAFs (PKH26^+^/PKH67^-^) as indicated in the corresponding quadrants as determined by flow cytometry (*left*) and quantification of these populations (*right*). **l** Phagocytic activity of CAFs toward PKH67^+^ ApoSQ (green) in the absence or presence of NSC23766. The nucleus and actin cytoskeleton of CAFs were stained with DAPI (blue) and TRITC (tetramethylrhodamine)-conjugated phalloidin (red), respectively. Representative image of CAFs phagocytosing ApoSQ (*left*). The ROI − Merge panels show high-magnification images of regions of interest as indicated by the white squares in the Merge panels. Original magnification: ×200. Scale bars = 20 μm. The *arrowheads* indicate CAFs with engulfed apoptotic 344SQ cells or fragments. Phagocytosis was quantified by a phagocytic index based on confocal microscopy (*right*). Immunoblot analysis of the indicated proteins in CAFs exposed to ApoSQ for 20 h (**b**, **e**, **m**) or 2 h (**h**). **c**, **f**, **i**, **n** ELISA of WISP-1 in CAF CM and ApoSQ-CAF CM 20 h after exposure to ApoSQ. Relative Rac1 activity in CAFs at the indicated time points after ApoSQ stimulation (**j**) or transfection with Notch1 siRNA before ApoSQ stimulation for 24 h (**o**). NS: not significant, *P < 0.05, **P < 0.01, ***P < 0.001, two-tailed Student’s *t* test. The data are from one experiment representative of three independent experiments with similar results (**a**, **d**, **g**, **k**, **l** and **p** *left*, **b**, **e**, **h**, **m**) or from three independent experiments (mean ± standard error; **a**, **d**, **g**, **k**, **l** and **p** *right*, **c**, **f**, **i**, **j**, **n**, **o**)

Brain-specific angiogenesis inhibitor 1 (BAI1) contributes to the efferocytosis of apoptotic cervical cancer cells by primary fibroblasts [20]. Thus, we investigated whether BAI1 signaling, which facilitates apoptotic lung cancer cell uptake [41], influences Notch1 signaling in CAFs. Several lines of evidence suggest a role for the thrombospondin type 1 repeats of BAI1 in direct phosphatidylserine (PtdSer) recognition and engulfment of apoptotic targets [42]. Thus, we first examined whether blocking the interaction between BAI1 in CAFs and ApoSQ through the addition of annexin V, which binds to PtdSer exposed on the surface of apoptotic cells, inhibits Notch1 signaling. Using quantitative flow cytometry, we measured the percentage of PKH26-stained CAFs that engulfed PKH67-stained ApoSQ after 24 h of coculture. Whereas the addition of annexin V inhibited the engulfment of ApoSQ by CAFs (Fig. 6a), Notch1 activation in and WISP-1 secretion from CAFs induced by ApoSQ were downregulated (Fig. 6b, c). Moreover, whereas knockdown of BAI1 (Supplementary Fig. S10a and Fig. 6d–f) or neutralization with an anti-BAI1 antibody (Supplementary Fig. S10b–d) attenuated the phagocytic activity of CAFs, Notch1 signaling and WISP-1 secretion were downregulated in CAFs treated with ApoSQ. In contrast, as the uptake rate of ApoSQ by BAI1-overexpressing CAFs was higher than that of mock-transfected cells (Supplementary Fig. S10e and Fig. 6g), BAI1 overexpression in CAFs further enhanced Notch1 signaling and WISP-1 secretion (Fig. 6h, i). Taken together, these results suggest that the ApoSQ binding protein BAI1 engages the Notch1 signaling pathway and thereby stimulates CAF WISP-1 production and efferocytosis.

We further examined the relationship between efferocytic activity and Notch1 signaling in CAFs using the Rac1 inhibitor NSC23766, which downregulates the phagocytic activity of apoptotic cells [42]. We found that exposure to ApoSQ enhanced Rac1 activity in CAFs in a time-dependent manner, with a peak at 24 h (Fig. 6j). Similar to the flow cytometry results (Fig. 6k), confocal microscopy confirmed the reduction in the phagocytic activity of CAFs by treatment with 100 μM NSC23766, which also reduced Notch1 signaling and WISP-1 secretion (Fig. 6l–n). Notch1 knockdown attenuated Rac1 activity (Fig. 6o), leading to reduced efferocytosis of ApoSQ by CAFs (Fig. 6p). Similarly, treatment with 20 μM DAPT inhibited Rac1 activity 24 h after exposure to ApoSQ (Supplementary Fig. S10f) and decreased engulfment of ApoSQ by CAFs (Supplementary Fig. S10g). Collectively, these results provide evidence of positive crosstalk between the Notch1 and BAI1-Rac1 pathways upon stimulation with apoptotic cancer cells.

### In vivo injection of ApoSQ enhances WISP-1 production and inhibits CAF activation and lung metastasis via Notch1 signaling

Our previous study demonstrated that a single injection of ApoSQ inhibited lung metastasis in syngeneic (129/Sν) immunocompetent mice [18]. Here, we used this mouse model to investigate the in vivo response of CAFs to injection of ApoSQ after subcutaneous injection of 344SQ cells (Supplementary Fig. S11a). We first isolated CAFs from primary tumors of mice using magnetic-activated cell sorting with Thy1 after removal of leukocytes, endothelial cells, and epithelial cells [24] and analyzed the mRNA levels of CAF markers using qRT‒PCR (Supplementary Fig. S11b, c) [24]. The mRNA expression levels of traditional CAF activation markers, including *Acta2, Col1α1, Fn, Itgβ1, Spp1, Pdgfrα, Pdgfrβ*, and *Mmp1a, 2, 9*, and *12*, and growth factors/chemokines, including *Vegfa, Hgf, Cxcl12, and Cxcl14*, were reduced after injection of ApoSQ. However, the mRNA expression levels of *Notch1* and Notch downstream target genes, including *Wisp1* (*Ccn4*)*, Hey1, Hey2, Hes1*, and *Hes5*, were increased after injection of ApoSQ. The mRNA expression levels of Notch ligands and members of the cellular communication network family, including *Ccn1, Ccn2, Ccn5*, and *Ccn6*, were unchanged after injection of ApoSQ.

**Fig. 7:**
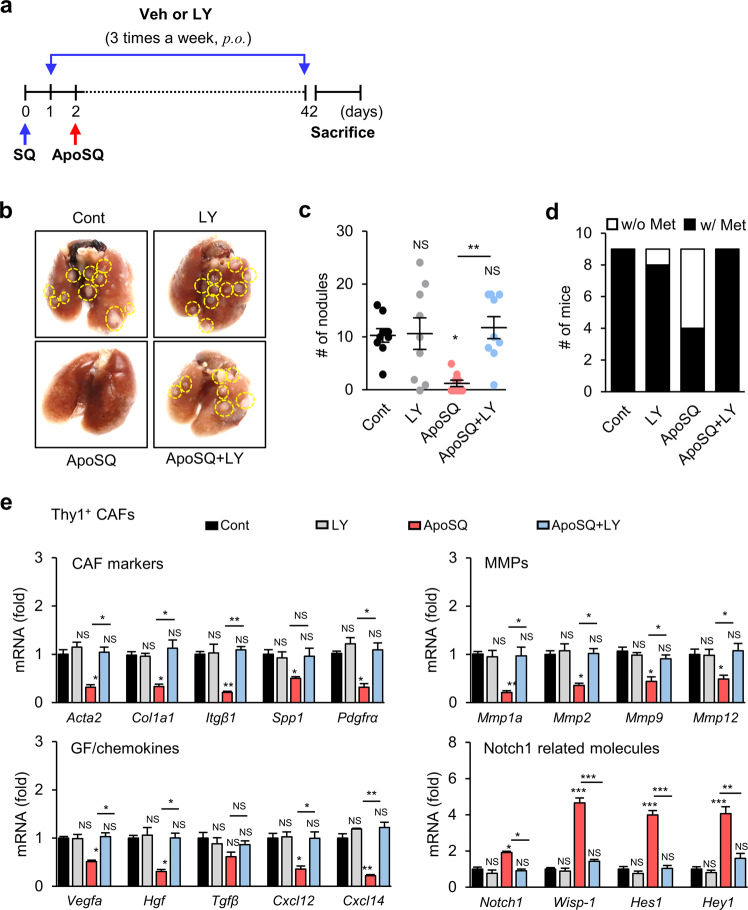
Notch1 signaling-dependent inhibitory effects of apoptotic lung cancer cell injection on CAF activation and metastasis in mice. **a** Schematic of the experimental design. Apoptotic 344SQ cells (ApoSQ) were subcutaneously injected into the established skin lesions 2 days after subcutaneous injection of 344SQ cells into syngeneic (129/Sν) mice (*n* = 9 per group). Where indicated, LY3039478 (8 mg/kg) or vehicle (Veh; 15% sugar gel) was orally administered three times a week for 6 weeks starting the day before injection of ApoSQ (*n* = 9 per group). Mice were necropsied 6 weeks after 344SQ cell injection. **b** Representative images of lungs with or without metastatic tumor nodules (yellow dashed circles). **c** Scatter plot of the numbers of lung metastatic nodules. NS: not significant, *P < 0.05, **P < 0.01, Kruskal‒Wallis test with Dunn’s post hoc test. The data are from representative images (**b**) or from independent experiments with 9 mice per group (mean ± standard error; **c**). **d** Numbers of mi**c**e with (w/) and without (w/o) visibly determined metastases (Met). **e** qRT‒PCR analysis of CAF markers, MMPs, growth factors (GFs)/chemokines, and Notch1-related molecules in Thy1^+^ CAFs isolated from primary tumors. NS: not significant, *P < 0.05, **P < 0.01, ***P < 0.001, Kruskal‒Wallis test with Dunn’s post hoc test. The data are from three replicates per condition, with cells pooled from three mice per replicate. The data are shown as the means ± standard errors

**Fig. 8:**
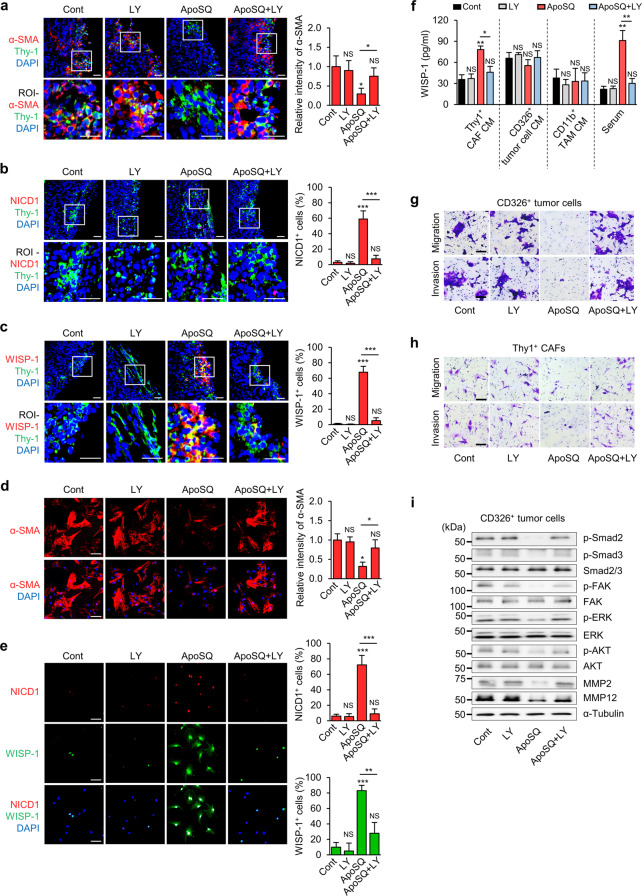
Inhibition of Notch1 signaling reverses the increase in WISP-1 production and the inhibition of migration, invasion and signaling pathways in vivo. The experimental design was as described in Fig. 7. **a**–**c** Representative confocal images of primary tumor sections stained with an anti-α-SMA antibody (red; **a**), an anti-NICD1 antibody (red; **b**), and an anti-WISP-1 antibody (red; **c**), an anti-Thy1 antibody (green), and DAPI (blue) (*left*). The ROI − Merge panels show high-magnification images of regions of interest as indicated by the white squares in the Merge panels. Original magnification: ×200. Scale bars = 20 μm. Quantification of α-SMA staining in Thy1^+^ cells and of NICD1^+^ cells and WISP-1^+^ cells among Thy1^+^ cells (*right*). The data are presented as the means ± standard errors from three mice per group (**a**–**c** *right*). **d**, **e** Representative confocal images of isolated Thy1^+^ CAFs from primary tumors stained with antibodies specific for α-SMA (red), NICD1 (red) or WISP-1 (green) (*left*). The imaging medium was VECTASHIELD fluorescent mounting medium containing DAPI. Images were acquired at ×200 magnification. Scale bars = 200 μm. Relative fluorescence intensity of α-SMA staining and quantification of NICD1^+^ cells and WISP-1^+^ cells (*right*). **f** ELISA of WISP-1 in the culture media of isolated Thy1^+^ CAFs, CD326^+^ tumor cells, and CD11b^+^ TAMs and in serum. Phase contrast microscopy of migrated and invaded CD326^+^ tumor cells (**g**) and Thy1^+^ CAFs (**h**). Scale bars: 100 µm. **i** Immunoblot analysis of proteins in isolated CD326^+^ tumor cells. NS: not significant, *P < 0.05, **P < 0.01, ***P < 0.001, Kruskal‒Wallis test with Dunn’s post hoc test. The data are from three replicates per condition, with cells pooled from three mice per replicate. The data are from one experiment representative of three independent experiments with similar results (**d** and **e** *left*, **g**–**i**) and are shown as the mean ± standard error from three independent experiments (**d** and **e** *right*, **f**)

To confirm the inhibitory role of Notch1-WISP-1 signaling in CAF activation and tumor progression in vivo, the Notch1-selective inhibitor LY3039478 (8 mg/kg) was administered orally three times a week for 6 weeks beginning the day before injection of ApoSQ (Fig. 7a). LY3039478 did not alter body weight, primary tumor weight or primary tumor volume compared with those in the control and ApoSQ groups (Supplementary Fig. S12a–d). However, LY3039478 reversed the reductions in the number of tumor nodules on the lung surface and metastasis rate induced by ApoSQ (Fig. 7b–d). LY3039478 also reversed the reductions in the mRNA expression levels of CAF markers, MMPs, growth factors, and chemokines and increases in the mRNA expression levels of Notch1 and Notch downstream target genes induced by ApoSQ in Thy1^+^ CAFs (Fig. 7e). Moreover, immunohistochemical analysis of serial sections of primary tumor tissue showed that LY3039478 reversed the reduction in α-SMA expression (Fig. 8a) and increases in NICD1 and WISP-1 expression in Thy1^+^ CAFs (Fig. 8b, c). Administration of LY3039478 with buffer had no such effects. Immunocytochemical analysis of Thy1^+^ CAFs isolated from primary tumors confirmed the alterations in protein expression (Fig. 8d, e). The increases in the WISP-1 protein level in Thy1^+^ CAF culture medium and serum from mice treated with ApoSQ were reversed in the ApoSQ + LY3039478 group (Fig. 8f). However, the levels of WISP-1 in the culture media of CD326^+^ tumor cells and CD11b^+^ TAMs were unchanged in the ApoSQ and ApoSQ + LY3039478 groups. The migration and invasion of CD326^+^ tumor cells and Thy1^+^ CAFs were reduced after injection of ApoSQ (Fig. 8g, h and Supplementary Fig. S12e, f). Likewise, injection of ApoSQ inhibited the activation of migration- and invasion-related signaling pathways, including the Smad2/3, FAK, ERK, and Akt pathways, as well as the protein expression of MMP2 and MMP12 in CD326^+^ tumor cells (Fig. 8i and Supplementary Fig. S12g). However, LY3039478 reversed the effects of injection of ApoSQ. Taken together, these results demonstrate that a single injection of ApoSQ inhibits the activation of CAFs but enhances Notch1-WISP-1 signaling in CAFs. In addition, targeting of Notch1-WISP-1 signaling in CAFs by injection of ApoSQ may play a critical role in the inhibition of CAF activation and metastasis.

### ApoSQ-CAF CM inhibits tumor progression and lung metastasis in vivo

**Fig. 9:**
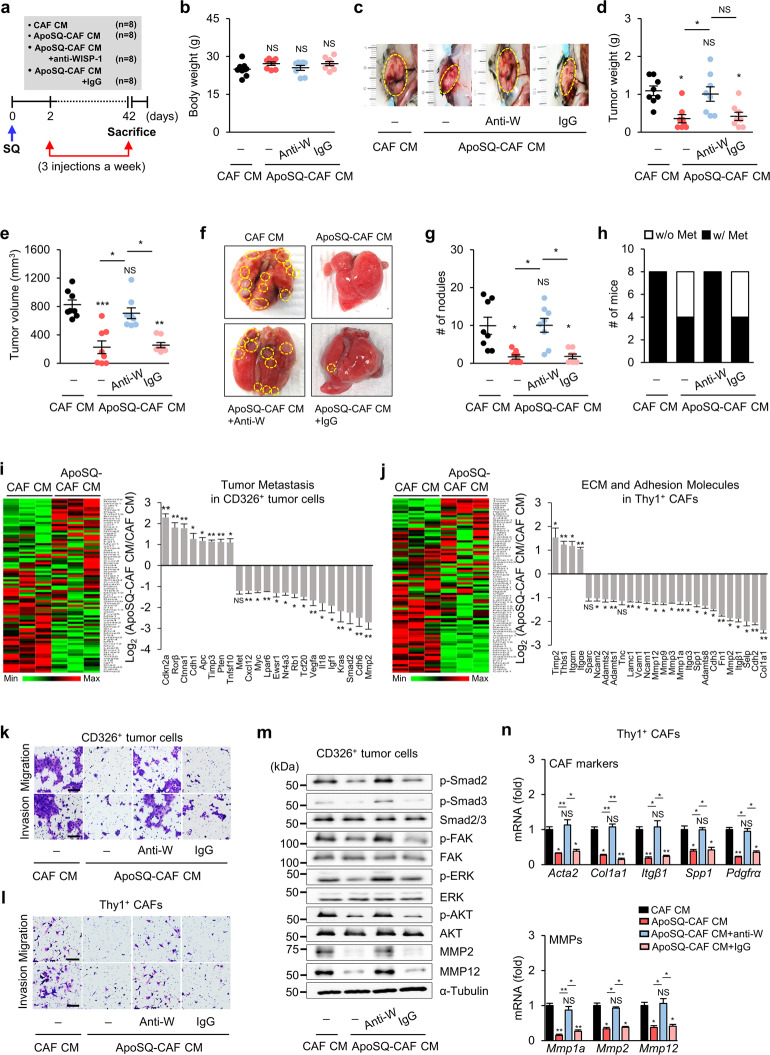
ApoSQ-CAF CM inhibits tumor growth and lung metastasis via WISP-1 in mice. **a** Schematic of the experimental design and group allocation. CAF CM, ApoSQ-CAF CM, ApoSQ-CAF CM + anti-WISP-1, or ApoSQ-CAF CM + IgG was intratumorally injected three times a week for 6 weeks starting 2 days after subcutaneous injection of 344SQ cells into syngeneic (129/Sν) mice (*n* = 8 mice per group). Mice were necropsied 6 weeks later. Scatter plots of body weight (**b**), primary tumor weight (**d**), tumor volume (**e**), and numbers of lung metastatic nodules (**g**). **c** Representative images of primary tumors (yellow dashed circles). **f** Representative images of lungs with and without metastases. The yellow dashed circles indicate lung metastatic nodules. NS: not significant, *P < 0.05, **P < 0.01, ***P < 0.001, Kruskal‒Wallis test with Dunn’s *post hoc* test. The data are presented as the mean ± standard error of results from 8 mice per group (**b**, **d**, **e**, **g**). **h** Numbers of mice with (w/) and without (w/o) visibly determined metastases (Met). **i** Heatmap showing differentially expressed tumor metastasis-related genes in CD326^+^ tumor cells isolated from primary tumors (*left*). Red: high expression; green: low expression. Relative expression of selected genes from PCR array profiling of tumor metastases (*right*). Log_2_ fold change values (ApoSQ-CAF CM vs. CAF CM). **j** Heatmap showing differentially expressed genes encoding ECM and adhesion molecules in Thy1^+^ CAFs isolated from primary tumors (*left*). Relative expression levels of selected genes from PCR array profiling (*right*). Log_2_ fold change values (ApoSQ-CAF CM vs. CAF CM). NS: not significant, *P < 0.05, **P < 0.01, two-tailed Student’s *t* test. The data are from three replicates per condition with cells pooled from two or three mice per replicate (mean ± standard error; **i** and **j** *right*). Phase contrast microscopy of migrated and invaded CD326^+^ tumor cells (**k**) and Thy1^+^ CAFs (**l**). Scale bars: 100 ^µ^m. **m** Immunoblot analysis of proteins in CD326^+^ tumor cells. **n** qRT‒PCR analysis of CAF markers and MMPs in isolated Thy1^+^ CAFs. NS: not significant, *P < 0.05, **P < 0.01, Kruskal‒Wallis test with Dunn’s post hoc test. The data are from three replicates per condition with cells pooled from two or three mice per replicate. The data are from one experiment representative of three independent experiments with similar results (**k–m**) or from three independent experiments (mean ± standard error; **n**)

To investigate the in vivo effect of ApoSQ-CAF CM on tumor progression and lung metastasis, CAF CM or ApoSQ-CAF CM was injected intratumorally three times a week into syngeneic mice starting 2 days after 344SQ cell injection (Fig. 9a). Although the body weight was similar among the groups (Fig. 9b), injection of ApoSQ-CAF CM reduced the primary tumor weight and volume compared with CAF CM (Fig. 9c–e). Similar to a single injection of ApoSQ [18], injection of ApoSQ-CAF CM reduced the number of tumor nodules on the lung surface and the metastasis rate (Fig. 9f–h).

To evaluate the antimetastatic effects of ApoSQ-CAF CM at the gene level in vivo, we used a Mouse Tumor Metastasis RT^2^ Profiler PCR Array to allow simultaneous determination of the expression of 84 genes implicated in invasion and metastasis. Injection of ApoSQ-CAF CM downregulated the expression of genes (by >2 fold) involved in tumor progression and invasion (fifteen genes, including *MMP2, Cdh6, Smad2, Kras*, and *Igf1*) and upregulated the expression of genes (by >2 fold) involved in suppression of tumor progression and invasion (eight genes, including *Cdkn2a, Rorβ*, and *Ctnna1*) in isolated CD326^+^ tumor cells (Fig. 9i). To obtain additional insights into the mechanisms by which ApoSQ-CAF CM reduces CAF invasion in vivo, we analyzed 84 genes involved in cell adhesion and ECM remodeling using a targeted qRT‒PCR array. Eight cell adhesion-related genes were downregulated by more than twofold in the ApoSQ-CAF CM group compared with the CAF CM group: *Cdh2*, *Selp*, *Itgβ1, Cdh3, Itga3, Ncam1, Vacm1*, and *Ncam2* (Fig. 9j). ECM remodeling-related genes, such as ECM components (four genes) and MMPs (eight genes), were also downregulated (<2-fold) in the ApoSQ-CAF CM group compared with the CAF CM group.

To confirm the key role of WISP-1 in the anti-tumor progression and antimetastatic effects of ApoSQ-CAF CM in vivo, an anti-WISP-1 neutralizing antibody or IgG isotype control was added to the CM for 2 h before injection into mice. The tumor weight, tumor volume, nodule number and metastasis rate were unchanged after injection of WISP-1-immunodepleted ApoSQ-CAF CM, but CM containing the IgG isotype control had effects similar to those of ApoSQ-CAF CM (Fig. 9c–h). Consistent with our in vitro findings, injection of ApoSQ-CAF CM inhibited the migration and invasion of CD326^+^ tumor cells and Thy1^+^ CAFs, whereas injection of WISP-1-immunodepleted ApoSQ-CAF CM did not have inhibitory effects (Fig. 9k, l and Supplementary Fig. S13a, b). The activation of migration- and invasion-related signaling pathways, including the Smad2/3, FAK, ERK, and Akt pathways, and the protein expression levels of MMP2 and MMP12 in CD326^+^ tumor cells were diminished by injection of ApoSQ-CAF CM, whereas injection of WISP-1-immunodepleted ApoSQ-CAF CM had no such inhibitory effects (Fig. 9m and Supplementary Fig. S13c). qRT‒PCR analysis of Thy1^+^ CAFs showed that injection of ApoSQ-CAF CM reduced the mRNA expression levels of the CAF activation markers *Acta2, Col1α1, Itgβ1, Spp1, Pdgfrα, Mmp1a, Mmp2*, and *Mmp12* (Fig. 9n). However, injection of WISP-1-immunodepleted ApoSQ-CAF CM had no such inhibitory effects.

**Fig. 10:**
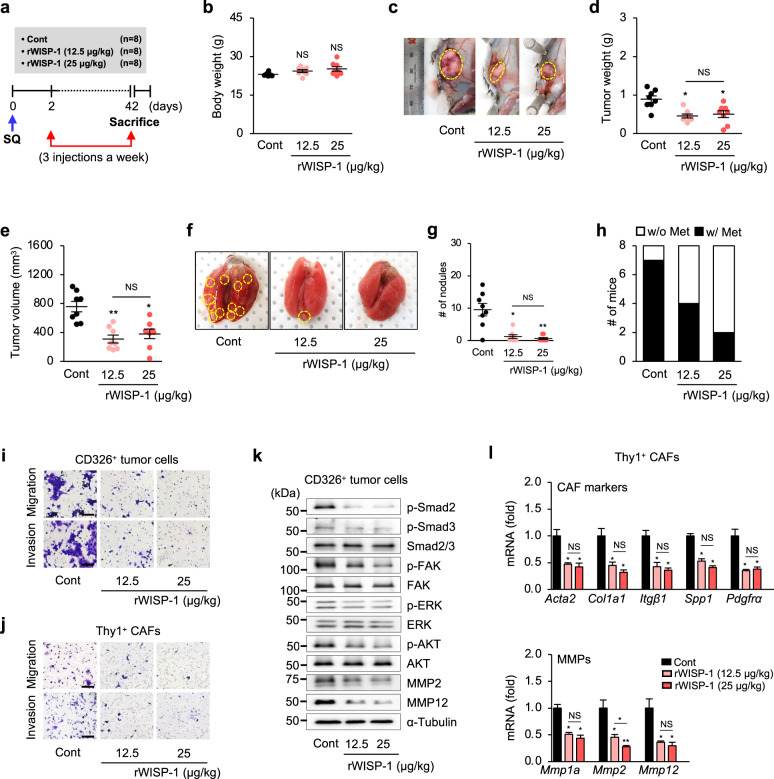
Recombinant WISP-1 inhibits tumor growth and lung metastasis in mice. **a** Schematic of the experimental design. Where indicated, rWISP-1 (12.5 and 25 μg/kg) was intratumorally injected three times a week for 6 weeks starting 2 days after subcutaneous injection of 344SQ cells into syngeneic (129/Sν) mice (*n* = 8 mice per group). Mice were necropsied 6 weeks later. Scatter plots of body weight (**b**), primary tumor weight (**d**), tumor volume (**e**), and numbers of lung metastatic nodules (**g**). **c** Representative images of primary tumors (yellow dashed circles). **f** Representative images of lungs with and without metastases. The yellow dashed circles indicate lung metastatic nodules. NS: not significant, *P < 0.05, **P < 0.01, Kruskal‒Wallis test with Dunn’s *post hoc* test. The data are presented as the mean ± standard error of results from 8 mice per group (**b**, **d**, **e**, **g**). **h** Numbers of mice with (w/) and without (w/o) visibly determined metastases (Met). Phase contrast microscopy of migrated and invaded CD326^+^ tumor cells (**i**) and Thy1^+^ CAFs (**j**). Scale bars^:^ 100 µm. **k** Immunoblot analysis of proteins in CD326^+^ tumor cells. **l** qRT^‒^PCR analysis of CAF markers and MMPs in isolated Thy1^+^ CAFs. NS: not significant, *P < 0.05, **P < 0.01, Kruskal‒Wallis test with Dunn’s post hoc test. The data are from three replicates per condition with cells pooled from two or three mice per replicate. The data are from one experiment representative of three independent experiments with similar results (**i–k**) or from three independent experiments (mean ± standard error; **l**)

In addition, to further confirm the anti-tumor progression and antimetastatic effects of WISP-1, rWISP-1 (12.5 and 25 μg/kg) was injected intratumorally three times a week into syngeneic mice starting 2 days after 344SQ cell injection (Fig. 10a). Similar to the inhibitory effects of ApoSQ-CAF CM injection, injection of rWISP-1 reduced the primary tumor weight and volume as well as the number of tumor nodules on the lung surface and metastasis rate compared with those in the control group (Fig. 10b–h). rWISP-1 inhibited the migration and invasion of CD326^+^ tumor cells and Thy1^+^ CAFs (Fig. 10I, j and Supplementary Fig. S14a, b). Notably, the inhibitory effects of rWISP-1 on the lung metastasis rate and cell migration and invasion were dose dependent. rWISP-1 also blocked signaling pathway activation and the protein expression of MMP2 and MMP12 in CD326^+^ tumor cells (Fig. 10k and Supplementary Fig. S14c) and reduced the mRNA expression levels of CAF activation markers in Thy1^+^ CAFs (Fig. 10l). Taken together, these results confirm that these tumor-antagonizing effects of ApoSQ-CAF CM are strongly mediated by WISP-1. In addition, rWISP-1 can fully mimic the in vivo effects of ApoSQ-CAF CM.

## Discussion

The results of this study advance our understanding of the important role of apoptotic lung cancer cells in reprogramming phagocytic CAFs and thereby inhibiting the migration and invasion of cancer cells and CAFs via paracrine and autocrine mechanisms. Our results showed that these antimigratory and anti-invasive effects were specific and not limited to lung cancer cells. Apoptotic bodies, vesicles, and soluble factors released from apoptotic tumor cells can influence tumor progression and fibroblast activation [43–45]. However, under our experimental conditions, this possibility can be ruled out, because the CM of ApoSQ or NecSQ alone did not affect the migration or invasion of 344SQ cells or CAFs. In addition, direct exposure of 344SQ cells to ApoSQ or NecSQ did not inhibit their migration or invasion. These results suggest that interaction between apoptotic cancer cells and CAFs plays a critical role in preventing their migration and invasion. Alba-Castellón and colleagues [46] showed that CAF activation controls epithelial tumor cell invasion and metastasis in an EMT-independent manner. They also reported that enhanced migration and invasion of CAFs via TGF-β/Snail1 signaling facilitates epithelial tumor cell invasion toward nutrient-rich territories [47]. Therefore, downregulating the migration and invasion abilities of CAFs could attenuate tumor progression and metastasis.

Elucidating the paracrine and autocrine mechanisms involved in CAF reprogramming is of great importance for the development of effective tumor-suppressive and antimetastatic therapies. In in vitro experiments employing various approaches, including cytokine array analysis, WISP-1 knockdown and overexpression, and WISP-1 immunodepletion, we found that WISP-1 secreted from CAFs after exposure to ApoSQ plays a critical role in inhibiting TGF-β1-induced migration and invasion of 344SQ cells and CAFs. Consistent with our observation of WISP-1 expression by CAFs, WISP1 overexpression in human lung cancer cells and mouse melanoma cells suppressed the migration and invasion of cancer cells in vitro, preventing the lung metastasis of murine melanoma cells in vivo [37, 48]. Lower WISP-1 expression was observed in breast cancer patients with poor prognosis, suggesting that intracellular WISP-1 expression acts as a tumor suppressor [49, 50]. In contrast, other groups have shown that WISP-1 knockout inhibits melanoma cell invasion and metastasis by suppressing EMT in mouse melanoma cells [51, 52]. A relatively high concentration of rWISP1 (5 μg/ml) restored EMT-associated gene expression [52]. However, in the present study, a lower concentration of rWISP-1 (≤10 ng/ml) had similar antimigratory and anti-invasive effects on 344SQ cells and CAFs. This discrepancy among studies might be explained by differences in cancer type and stage, the concentration of WISP-1 in the TME and/or the cellular context. Considering the complexity of WISP-1 interactions with multiple protein partners as well as the regulation of their expression, it is not yet clear how WISP-1 modulates cellular functions to exert oncogenic or tumor-suppressive effects in different tumor types [51]. Notably, we demonstrated that consistent with the effects of exposure to ApoSQ-CAF CM and ApoSQ, treatment with rWISP-1 blocked Smad-dependent and Smad-independent TGFβ1 signaling pathways and inhibited MMP2 and MMP12 mRNA and protein expression in 344SQ cells and CAFs. These findings suggest that WISP-1 secretion in the TME prevents the migration and invasion of lung cancer cells and CAFs by inhibiting multiple TGFβ1 signaling pathways. Furthermore, based on experiments using blocking antibodies against integrin α5, αv, β1, β3, or β5, our data suggest that integrin ανβ3 in 344SQ cells and integrin ανβ5 in CAFs act as functional receptors for WISP-1.

Stromal fibroblasts, in which the Notch1 pathway is constitutively activated, attenuate melanoma growth and suppress tumor angiogenesis partially by upregulating WISP-1 [11]. A more recent study using mesenchymal stem cell-derived fibroblasts suggests that intracellular Notch1 signaling in CAFs is a molecular switch that controls the plasticity and stemness of melanoma stem/initiating cells [53]. In the present study, we discovered a novel mechanism by which apoptotic lung cancer cells induce CAF reprogramming to promote a tumor-suppressive environment by activating Notch1 signaling, leading to transcriptional upregulation of WISP-1. Notch signaling is induced primarily by a family of delta/serrate/LAG-2 ligands [40]. We found that among various Notch ligands on UV-irradiated apoptotic cancer cells, Dll1 exhibited enhanced expression, suggesting that an interaction between Dll1 on the surface of apoptotic cancer cells and the Notch1 receptor on CAFs is responsible for most Notch signaling. Indeed, treatment with an anti-Dll1 blocking antibody or transfection with Dll1 siRNA downregulated Notch1-WISP-1 signaling in CAFs following ApoSQ stimulation. Although the traditional paradigm of Notch activation focuses on Notch receptor‒ligand interactions, it is becoming increasingly clear that Notch signaling is influenced by a wide array of molecules and events in the cellular microenvironment [54, 55]. Thus, various ECM molecules and other signaling mechanisms regulate Notch signaling via direct interactions with Notch receptors or ligands in the cellular microenvironment.

Park et al. [42] showed that thrombospondin type 1 repeats within the extracellular region of BAI1 mediate the direct binding of BAI1 to PtdSer. BAI1 cooperates with ELMO/Dock189/Rac to promote the engulfment of apoptotic cells. In the present study, we found that the BAI1/Rac1 signaling pathway positively controls Notch1 signaling, leading to WISP-1 secretion from and efferocytosis of apoptotic lung cancer cells by CAFs. Annexin V treatment, BAI1 knockdown, and anti-BAI1 blocking antibody treatment partially inhibited Notch1 signaling activation and WISP-1 secretion induced by ApoSQ. Conversely, overexpression of BAI1 in CAFs reinforced Notch1 signaling induced by ApoSQ via WISP-1 secretion. Rac1 inhibition by NSC23766 had a negative effect on Notch1 signaling and WISP-1 secretion. On the other hand, inhibition of Notch1 signaling by DAPT or transfection with Notch1 siRNA downregulated Rac1 activity and reduced the efferocytic ability of CAFs. These findings suggest that crosstalk between the BAI1/Rac1 and Notch1 signaling pathways is pivotal for maintaining the optimal CAF efferocytic ability and concurrent WISP-1 production. Additional studies are needed to further delineate and define the contributions of other phagocytic receptors, extracellular bridging molecules, and efferocytosis-related signaling molecules that participate in crosstalk with the Notch1 signaling pathway not only in CAFs but also in professional phagocytes in the TME.

Using a syngeneic mouse model, we demonstrated here that injection of ApoSQ targeted and inhibited the activation of CAFs, as evidenced by the reduced mRNA levels of multiple CAF activation markers, including those encoding ECM proteins, MMPs, and growth factors/chemokines, but enhanced the expression of Notch1 downstream target genes in Thy1^+^ CAFs isolated from primary tumors. Additionally, immunofluorescence analysis of primary tumor tissue and isolated Thy1^+^ CAFs confirmed the reduced α-SMA expression and increased NICD1 and WISP-1 expression in Thy1^+^ CAFs after injection of ApoSQ. Interestingly, the WISP-1 level was increased in culture medium of Thy1^+^ CAFs and in serum but not in culture medium of CD326^+^ tumor cells or CD11b^+^ TAMs isolated from primary tumors, indicating that the source of WISP-1 was CAFs in the TME. Notably, injection of ApoSQ changed Thy1^+^ CAF and CD326^+^ tumor cell phenotypes, attenuating their migratory and invasive activities and inactivating related signaling pathways. The overall effects of the Notch1-selective inhibitor LY3039478 on reversing these phenotypes suggest the critical role of Notch1-WISP-1 signaling in CAFs targeted by injected ApoSQ in the induction of inhibitory effects on CAF activation and metastasis in vivo.

Importantly, injection of ApoSQ-CAF CM after 344SQ cell injection reduced primary tumor size and volume. However, these effects were not observed in mice injected with ApoSQ [18]. The number of metastatic nodules and the metastasis rate were likely diminished by injection of ApoSQ-CAF CM. In addition, qPCR array analysis revealed downregulation of genes involved in tumor progression and invasion and upregulation of genes involved in the suppression of tumor progression and invasion in CD326^+^ tumor cells after injection of ApoSQ-CAF CM, consistent with its antimetastatic effect. Similarly, gene expression patterns in Thy1^+^ CAFs, including downregulation of genes related to cell-ECM adhesion and ECM remodeling, were similar to those in noninvasive fibroblasts [56]. Consistent with these findings, the migration and invasion abilities of isolated Thy1^+^ CAFs and CD326^+^ tumor cells, as well as the expression of activation markers of the related signaling pathways in CD326^+^ tumor cells and of CAFs, were attenuated by injection of ApoSQ-CAF CM. Importantly, these inhibitory effects were reversed by injection of WISP-1-immunodepleted ApoSQ-CAF CM. Similarly, intratumoral injection of rWIPS-1 resulted in these antitumorigenic and antimetastatic effects. Collectively, these in vivo results strongly suggest the key role of WISP-1 in ApoSQ-CAF CM in attenuating tumor growth and the migration and invasion of tumor cells and CAFs, thus preventing the development of metastases.

In summary, we propose that UV-irradiated apoptotic lung cancer cells trigger Notch1-WISP-1 signaling in CAFs, thereby preventing the migration and invasion of cancer cells and CAFs. The BAI1/Rac1 and Notch1 signaling pathways participate in positive crosstalk to maintain the optimal CAF efferocytic ability and concurrent WISP-1 production. Consistent with this finding, in vivo data suggest that a single injection of ApoSQ targets CAFs and enhances Notch1-WISP-1 signaling, leading to a less-invasive CAF phenotype and attenuating lung metastasis. Furthermore, administration of ApoSQ-CAF CM or rWIPS-1 inhibits tumor growth, the migration and invasion of tumor cells and CAFs, and the development of metastases. These data confirm our hypothesis that the tumor-antagonizing effects of ApoSQ-CAF CM are strongly mediated by WISP-1. Thus, apoptotic cancer cell therapies targeting CAFs or cell-free therapies, such as treatment with CM from CAFs exposed to apoptotic cancer cells and CM components such as WISP-1, could be effective therapeutic approaches to inhibit lung cancer progression and metastasis.

## Supplementary information

Supplementary materials
