# Supplementary material for: Reprogramming of cancer-associated fibroblasts by apoptotic cancer cells inhibits lung metastasis via Notch1-WISP-1 signaling
Source: Cell Mol Immunol. 2022 Oct 14;19(12):1373–91. doi: 10.1038/s41423-022-00930-w (PMC9708692; doi:10.1038/s41423-022-00930-w)
Supplement: Supplementary file 1 — Supplementary materials [file 41423_2022_930_MOESM1_ESM.pdf]

Supplementary material

Reprogramming of cancer-associated fibroblasts by apoptotic cancer cells inhibits lung metastasis via Notch1-WISP-1 signaling

Hee Ja Kim, Kyungwon Yang, Kiyoon Kim, Ye-JI Lee, Sieun Lee, Sung Yong Ahn, Young-Ho Ahn, and Jihee Lee Kang*

*Correspondence should be addressed to:

jihee@ewha.ac.kr

Supplementary Figures

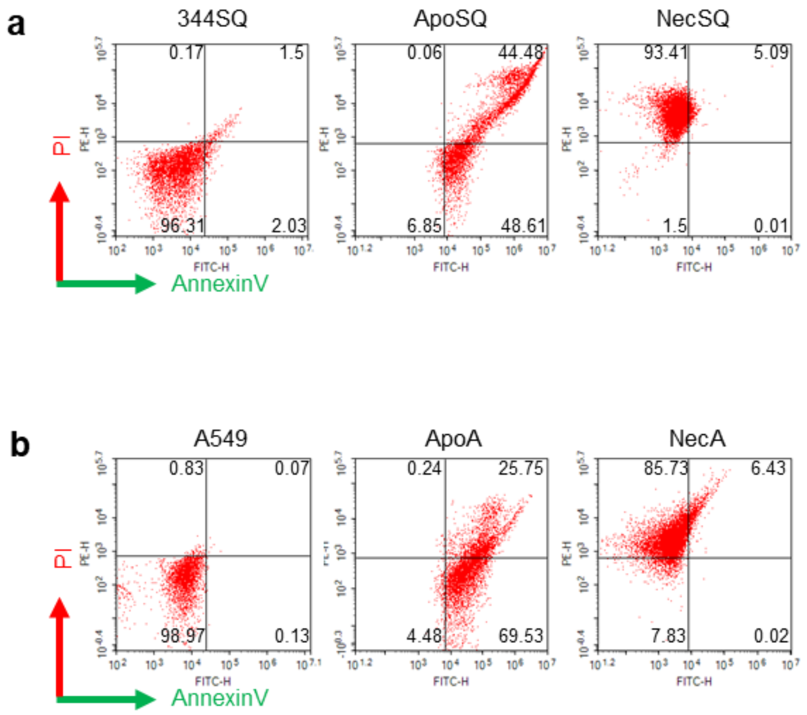

Supplementary Fig. S1. Representative dot plots depicting percentage of 344SQ and A549 cells. Numbers within quadrants represent the percentage of cells within each quadrant. (**a, b**) Early apoptotic cells (annexin positive and propidium iodide negative) appear in the lower right quadrant. Late apoptotic cells (positive both annexin and propidium iodide) appear in the upper right quadrant. (**a**) 344SQ and (**b**) A549 cells were UV irradiated for 15 min followed by incubation in RPMI-1640 with 10% FBS for 2 h at 37°C and 5% CO_2. Necrotic cells (propidium iodide positive) appear in the upper left quadrant. Lysed (necrotic) cancer cells were obtained by multiple freeze-thaw cycles. Cells were stained with annexin-V and propidium iodide to discriminate between apoptotic and necrotic cell death.

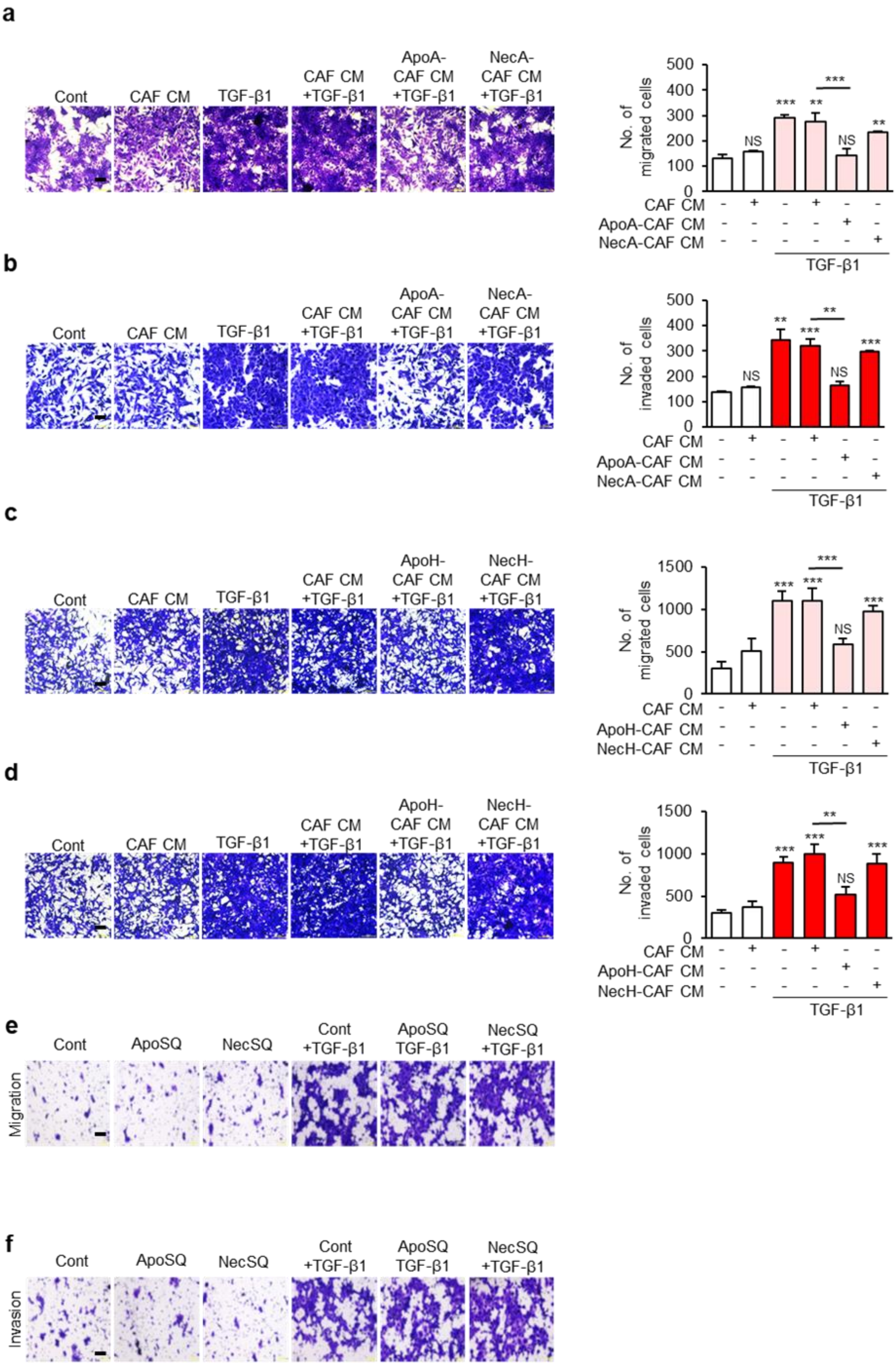

Supplementary Fig. S2. Interaction between CAFs and apoptotic cancer cells inhibits migration and invasion of cancer cells. (**a-d**) Phase-contrast microscopy (*left*) and

quantification of migrated and invaded cancer cells (*right)*, including A549 cells (**a**, **b**) and HCT116 cells (**c**, **d**). CAFs were exposed to apoptotic A549 (ApoA) and HCT116 (ApoH) or necrotic cancer cells (NecA and NecH) for 20 h. CM from CAFs was added to corresponding cancer cells with TGF-β1 (10 ng/ml) for 48 h. Phase-contrast microscopy of migrated (**e**) and invaded 344SQ cells (**f**). 344SQ cells were directly exposed to ApoSQ or NecSQ for 20 h and then replaced with fresh medium with or without TGF-β1 for 48 h. (**a**-**f**) Scale bars: 100 μm. NS: not significant; $^{**}P < 0.01$, $^{***}P < 0.001$, two-tailed Student's *t*-test. Data are from one experiment representative of three independent experiments with similar results (**a**-**d** ***left***, **e**, **f**), or from three independent experiments (mean ± standard error; **a**-**d** ***right***).

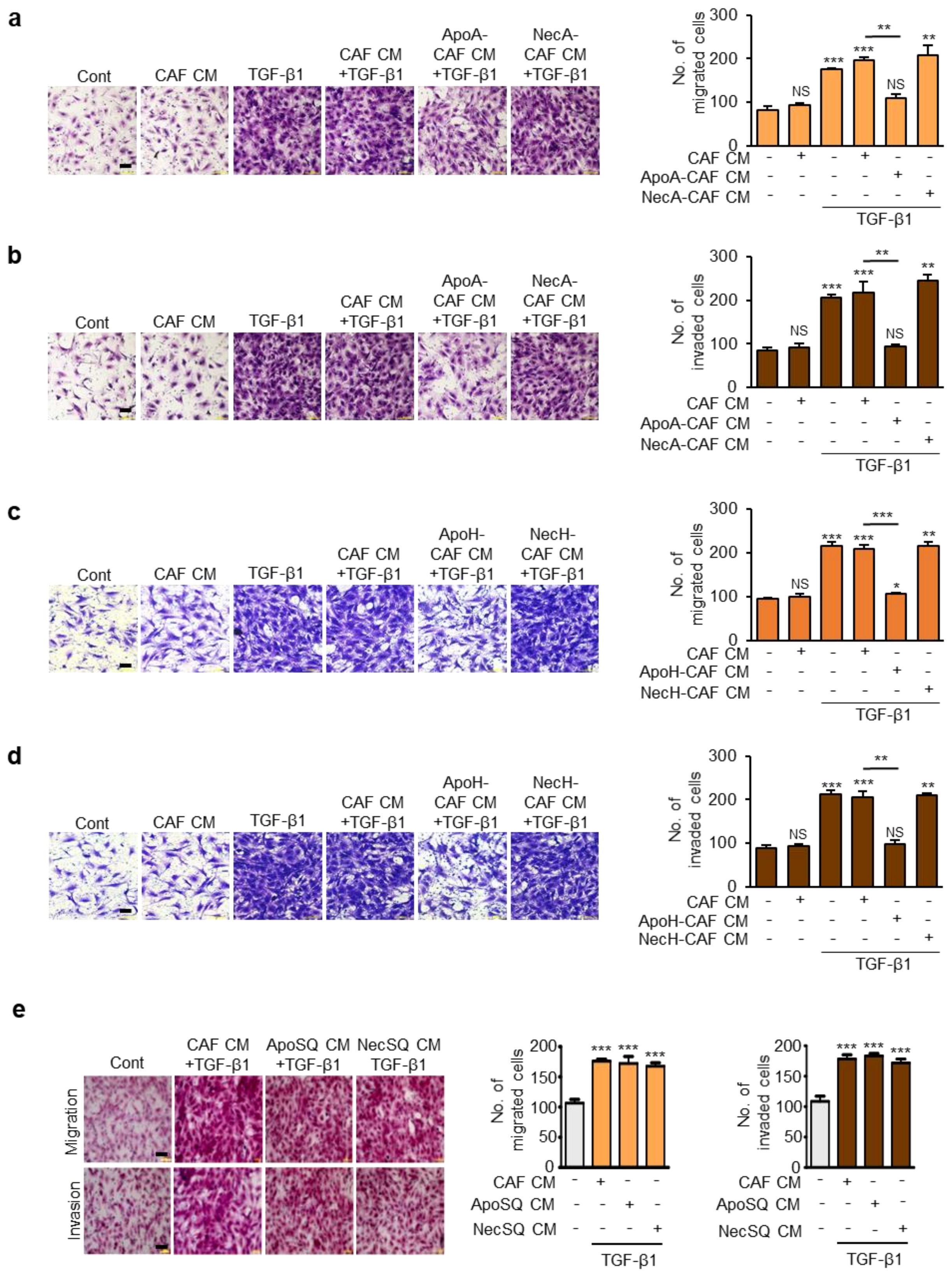

Supplementary Fig. S3. Interaction of CAFs and apoptotic lung cancer cells inhibits TGF-β1-induced migration, invasion, and activation markers in CAFs. (**a-e**) Phase-contrast

microscopy (*left*) and quantification of migrated and invaded CAFs (*right*). CAFs were exposed to ApoA, NecSQ (**a**, **b**), or ApoH, NecH (**c, d**) for 20 h. CM was added to CAFs with or without TGF-β1 (10 ng/ml) for 24 h. (**e**) ApoSQ CM or NecSQ CM was added to CAFs with TGF-β1 (10 ng/ml) for 24 h. (**a-f**) Scale bars: 100 μm. NS, not significant; **P* < 0.05, ***P* < 0.01, ****P* < 0.001, two-tailed Student's *t* test. Data are from one experiment representative of three independent experiments with similar results (**a**-**e** ***left***), or three independent experiments (mean ± standard error; **a**-**e** ***right***).

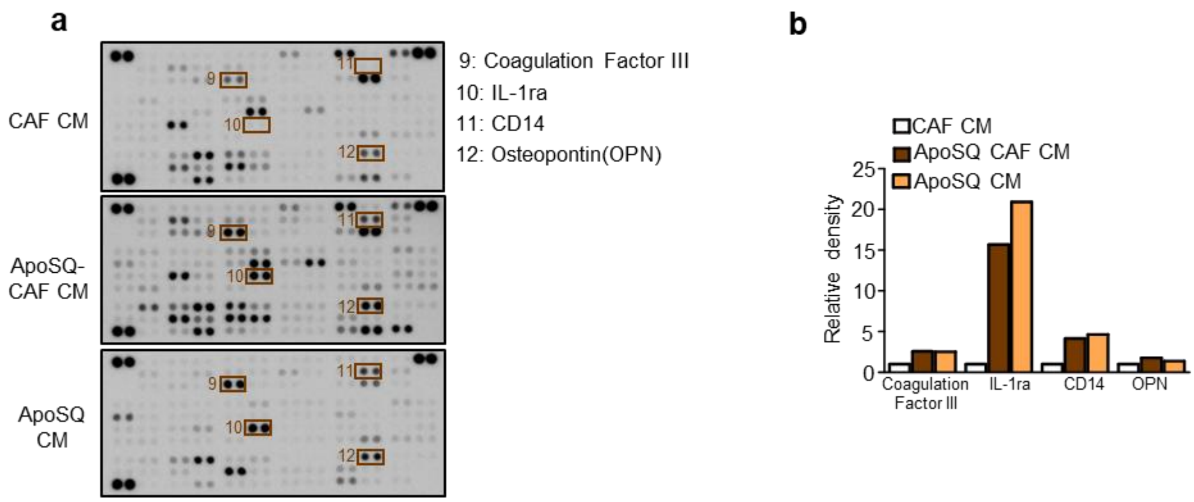

Supplementary Fig. S4. Cytokines in ApoSQ-CAF CM were comparable or less to their levels in ApoSQ CM. (**a**) Cytokine array of CAF CM, ApoSQ-CAF CM, or ApoSQ CM for 24 h. (**b**) Relative image densities of cytokine spots in ApoSQ-CAF CM and ApoSQ CM compared with CAF CM.

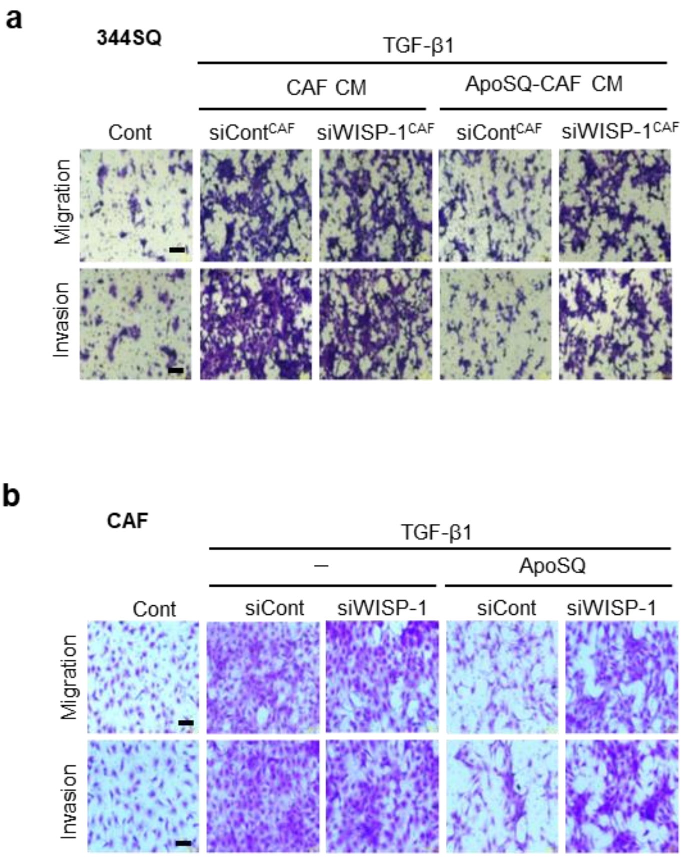

Supplementary Fig. S5. WISP-1 knockdown reverses the reduction of migration and invasion of 344SQ cells and CAFs. (**a**) Phase-contrast microscopy of migrated and invaded 344SQ cells. CAFs were transfected with control or WISP-1 siRNA before exposure to ApoSQ for 20 h. CM was added to 344SQ cells with TGF-β1 (10 ng/ml) for 48 h. (**b**) Phase-contrast microscopy of migrated and invaded CAFs. CAFs were transfected with WISP-1 siRNA before exposure to ApoSQ, and then replaced with fresh medium with TGF-β1 (10 ng/ml) for 24 h. (**a**, **b**) Scale bars: 100 μm. Data are from one experiment representative of three independent experiments with similar results (**a**, **b**).

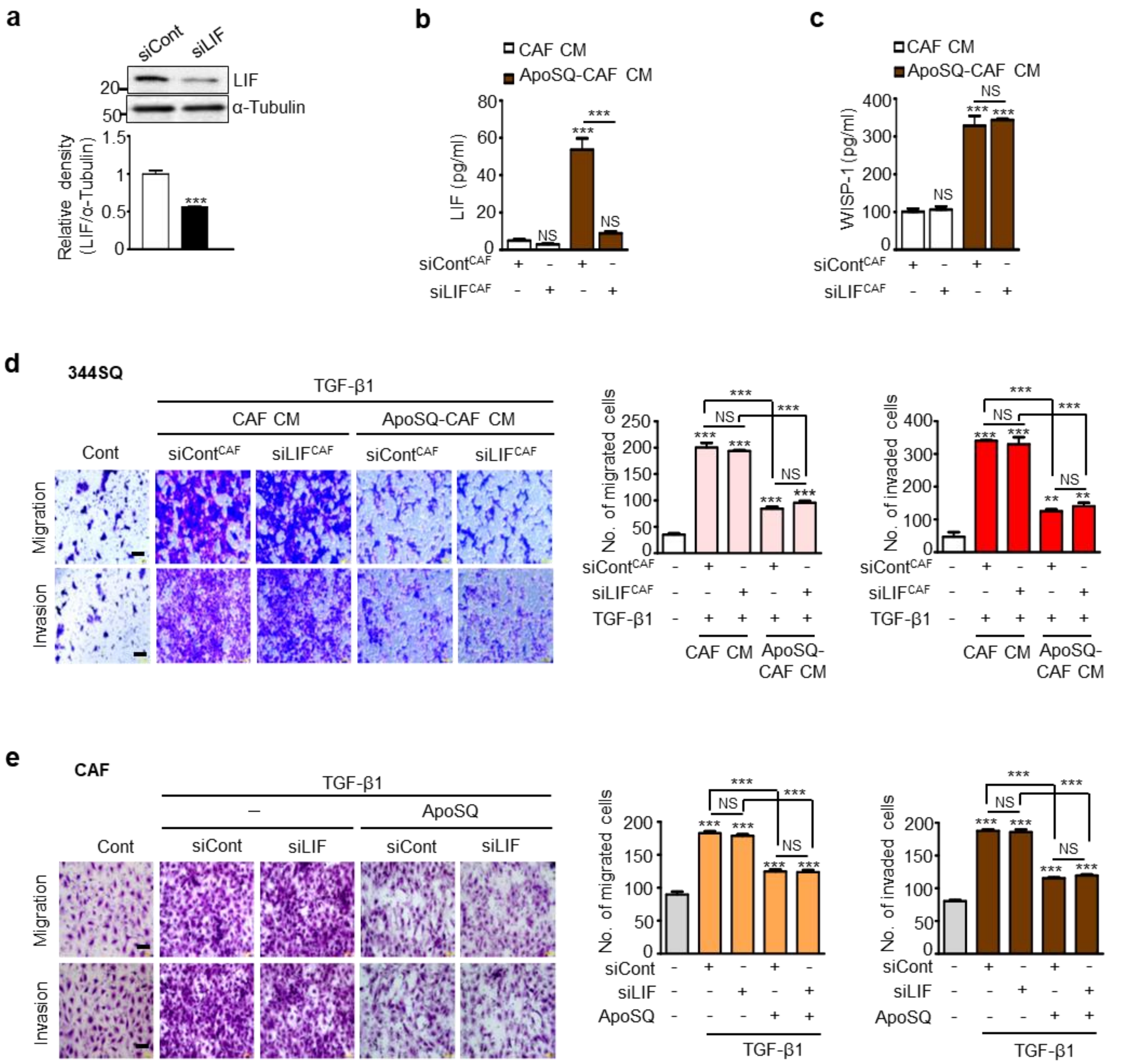

Supplementary Fig. S6. LIF secreted by CAFs does not affect migration and invasion of 344SQ cells and CAFs. (**a**) Immunoblot analysis of LIF in CAFs transfected with LIF siRNA (*upper*). Densitometric analysis of LIF relative abundances (*lower*). ELISA of LIF (**b**) and WISP-1 (**c**) in CAF CM and ApoSQ-CAF CM. CAFs transfected with control or LIF siRNA before exposure to ApoSQ for 20 h. (**d**) Phase-contrast microscopy (*left*) and quantification of migrated and invaded 344SQ cells (*right*). CAFs were transfected with control or LIF siRNA before exposure to ApoSQ. CM was added to 344SQ cells with TGF-β1 (10 ng/ml) for 48 h. (**e**) Phase-contrast microscopy (*left*) and quantification of migrated and invaded CAFs (*right*). CAFs were transfected with control or LIF siRNA before exposure to ApoSQ, and then replaced with fresh medium with TGF-β1 (10

ng/ml) for 24 h. (**d**, **e**) Scale bars: 100 μm. NS, not significant; ***P* < 0.01, ****P* < 0.001, two-tailed Student's *t* test. Data are from one experiment representative of three independent experiments with similar results (**a** ***upper***, **d** and **e** ***left***), or three independent experiments (mean ± standard error; **a** ***lower***, **b**, **c**, **d** and **e** ***right***).

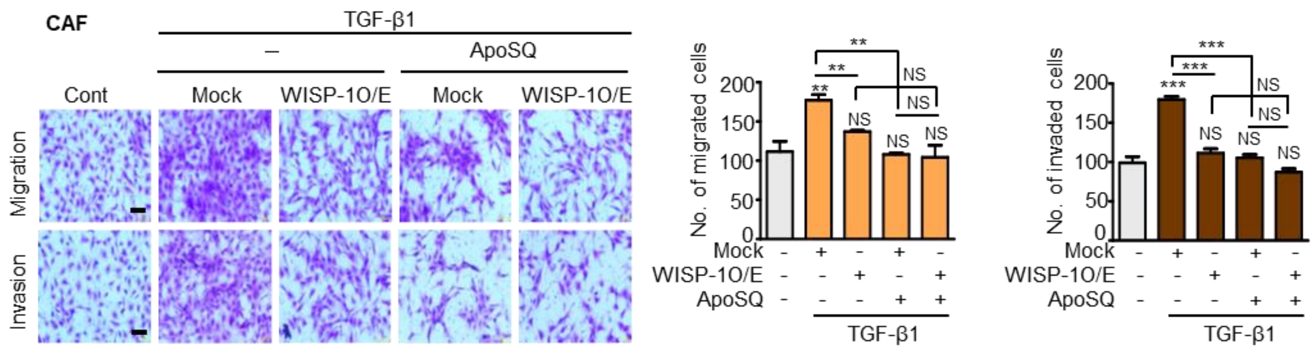

Supplementary Fig. S7. Migration and invasion of CAFs are regulated by modulating WISP-1 expression or activity. (**a**) Phase-contrast microscopy (*left*) and quantification of migrated and invaded CAFs (*right*). CAFs were transfected with empty vector or WISP-1 before treatment with or without ApoSQ for 20 h, and then replaced with fresh medium with TGF-β1 (10 ng/ml) for 24 h. Scale bars: 100 μm. NS, not significant; $^{**}P < 0.01$, $^{***}P < 0.001$, two-tailed Student's *t* test. Data are from three independent experiments (mean ± standard error; **a *right***), or one experiment representative of three independent experiments with similar results (**a *left***).

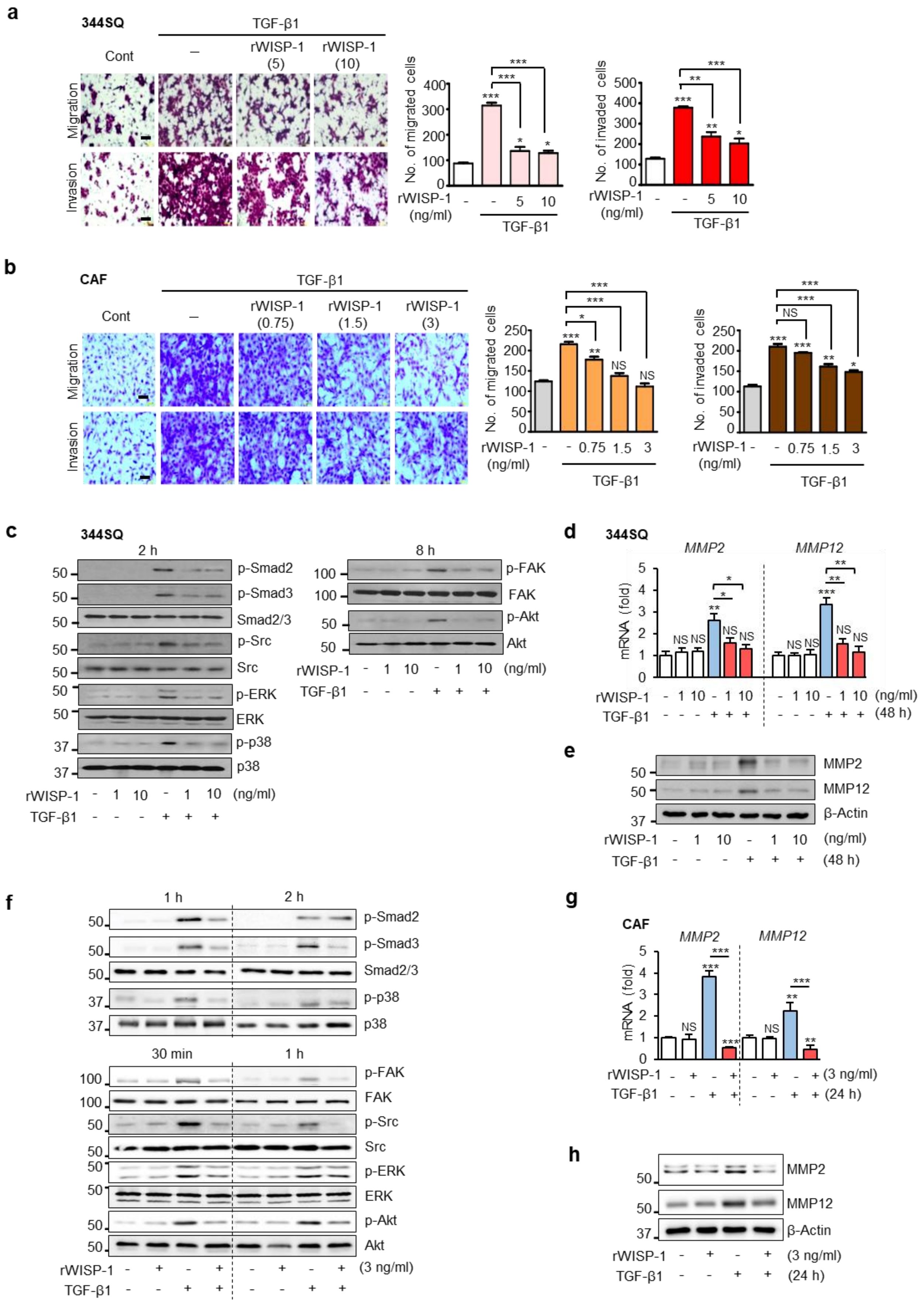

Supplementary Fig. S8. Recombinant WISP-1 treatment suppresses migration and invasion

of 344SQ cells and CAFs via blocking TGFβ1-induced signaling pathways. (**a**) Phase-contrast microscopy (*left*) and quantification of migrated and invaded 344SQ cells (*right)*. 344SQ cells were treated with rWISP-1 (5 and 10 ng/ml) with TGF-β1 (10 ng/ml) for 48 h. (**b**) Phase-contrast microscopy (*left*) and quantification of migrated and invaded CAFs (*right)***.** CAFs were treated with WISP-1 (0.75, 1.5, and 3 ng/ml) with TGF-β1 for 24 h. Scale bars: 100 μm. Immunoblot analysis of the indicated proteins in 344SQ (**c**, **e**) and CAF lysates (**f**, **h**) qRT-PCR analysis of *MM2* and *MMP12* mRNA in 344SQ (**d**) and CAF samples (**g**). (**c-h**). 344SQ cells and CAFs were directly exposed to rWISP-1 (1 and 10 ng/ml for 344SQ cells, 3 ng/ml for CAFs) with or without TGF-β1 (10 ng/ml) for the indicated time. NS: not significant; *$P < 0.05$, **$P < 0.01$, ***$P < 0.001$, two-tailed Student's *t*-tests. Data are from one experiment representative of three independent experiments with similar results (**a** and **b** ***left***, **c**, **e**, **f**, **h**), or three independent experiments (mean ± standard error; **a** and **b** ***right***, **d**, **g**).

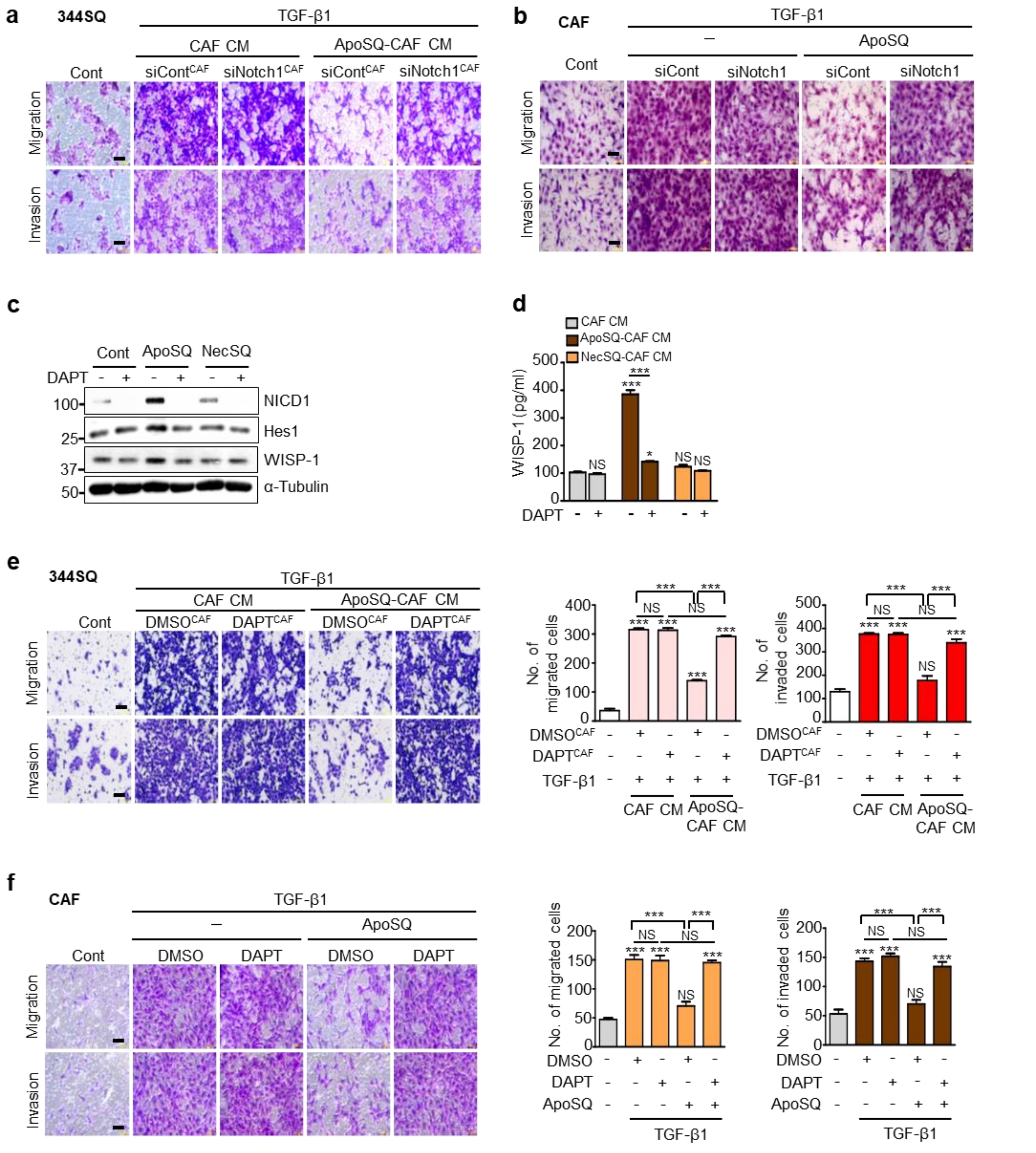

Supplementary Fig. S9. Notch 1 inhibition reverses anti-migration and -invasion effects. (**a**) Phase-contrast microscopy of migrated (*upper*) and invaded 344SQ (*lower*). CAFs were transfected with control or Notch1 siRNA before stimulation with ApoSQ. CM was added to 344SQ cells with TGF-β1 (10 ng/ml) for 48 h. (**b**) Phase-contrast microscopy of migrated (*upper*) and invaded CAFs (*lower*). CAFs were transfected with control or Notch1 siRNA before stimulation with

ApoSQ, and then replaced with fresh medium with TGF-β1 (10 ng/ml) for 24 h. (**c**) Immunoblot analysis of the indicated proteins in CAF lysates (left). (**d**) ELISA of WISP-1 in CAF CM, ApoSQ-CAF CM, and NecSQ-CAF CM. (**c**, **d**) CAFs were treated with DAPT (10 μM) before exposure to ApoSQ or NecSQ for 20 h. (**e**) Phase-contrast microscopy (*left*) and quantification of migrated and invaded 344SQ cells (*right)*. CAFs were treated with DAPT (10 μM) before exposure to ApoSQ. CM was added to 344SQ cells with TGF-β1 (10 ng/ml) for 48 h. (**f**) Phase-contrast microscopy (*left*) and quantification of migrated and invaded CAFs (*right)***.** CAFs were treated with DAPT (10 μM) before exposure to ApoSQ for 20 h, and then replaced with fresh media with TGF-β1 (10 ng/ml) for 24 h. (**d**, **e**, **h**, **i**) Scale bars: 100 μm. NS, not significant; **P* < 0.05, ***P* < 0.01, ****P* < 0.001, two-tailed Student's *t* test. Data are from one experiment representative of three independent experiments with similar results (**a**, **b**, **c, e** and **f** ***left***) or three independent experiments (mean ± standard error; **d**, **e** and **f** ***right***).

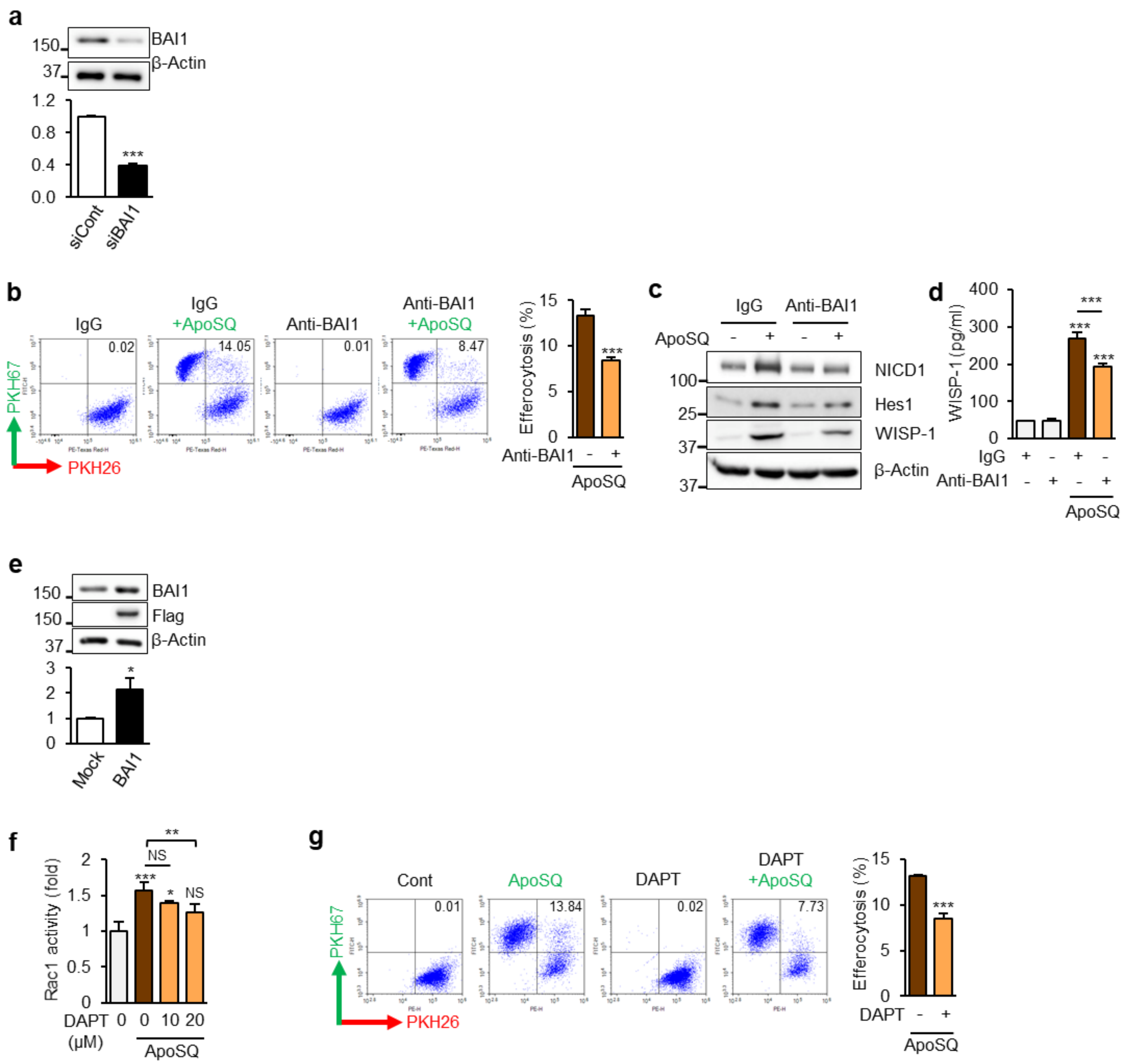

Supplementary Fig. S10. BAI1/Rac1 signaling positively crosstalks with Notch1-WISP1 signaling in CAFs. (**a**) Immunoblot analysis of BAI1 in CAFs transfected with control or BAI1 siRNA (*uppe*r). Densitometric analysis of the relative abundances of BAI1 (*lower*). (**b**) Phagocytic activity of ApoSQ by CAFs exposed to ApoSQ for 24 h with anti-BAI1 antibodies (8 μg/ml) or isotype IgG. Representative dot plots showing percentages of phagocytosing CAFs (PKH26$^+$/PKH67$^+$) and non-phagocytosing CAFs (PKH26$^+$/PKH67$^-$) as indicated in their respective quadrants as determined by flow cytometry (*left*) and their quantification (*right*). (**c**) Immunoblot analysis of the indicated proteins in CAFs exposed to ApoSQ for 20 h with anti-BAI1 antibodies (8 μg/ml) or isotype IgG. (**d**) ELISA of WISP-1 in CAF CM and ApoSQ-CAF CM at 20 h after

exposure to ApoSQ. (**e**) Immunoblot analysis of BAI1 and Flag proteins in CAFs transfected with control vehicle (Mock) or BAI1-Flag for 24 h (*upper*). Densitometric analysis of the relative abundances of BAI1 (*lower*). (**f**) Relative Rac1 activity in CAFs at 24 h after exposure to ApoSQ in the absence or presence of DAPT (10 and 20 μM). (**g**) Phagocytic activity of ApoSQ by CAFs in the absence or presence of 20 μM DAPT. Representative dot plots for CAFs phagocytosing ApoSQ determined by flow cytometry (*left*). Quantitative analysis of ApoSQ engulfment by CAFs (right). NS, not significant; $^{*}P < 0.05$, $^{**}P < 0.01$, $^{***}P < 0.001$, two-tailed Student's *t* test. Data are from one experiment representative of three independent experiments with similar results (**a** and **e** ***upper***, **b** and **g** ***left***, **c**) or three independent experiments (mean ± standard error; **a** and **e** ***lower***, **b** and **g** ***right***, **f**).

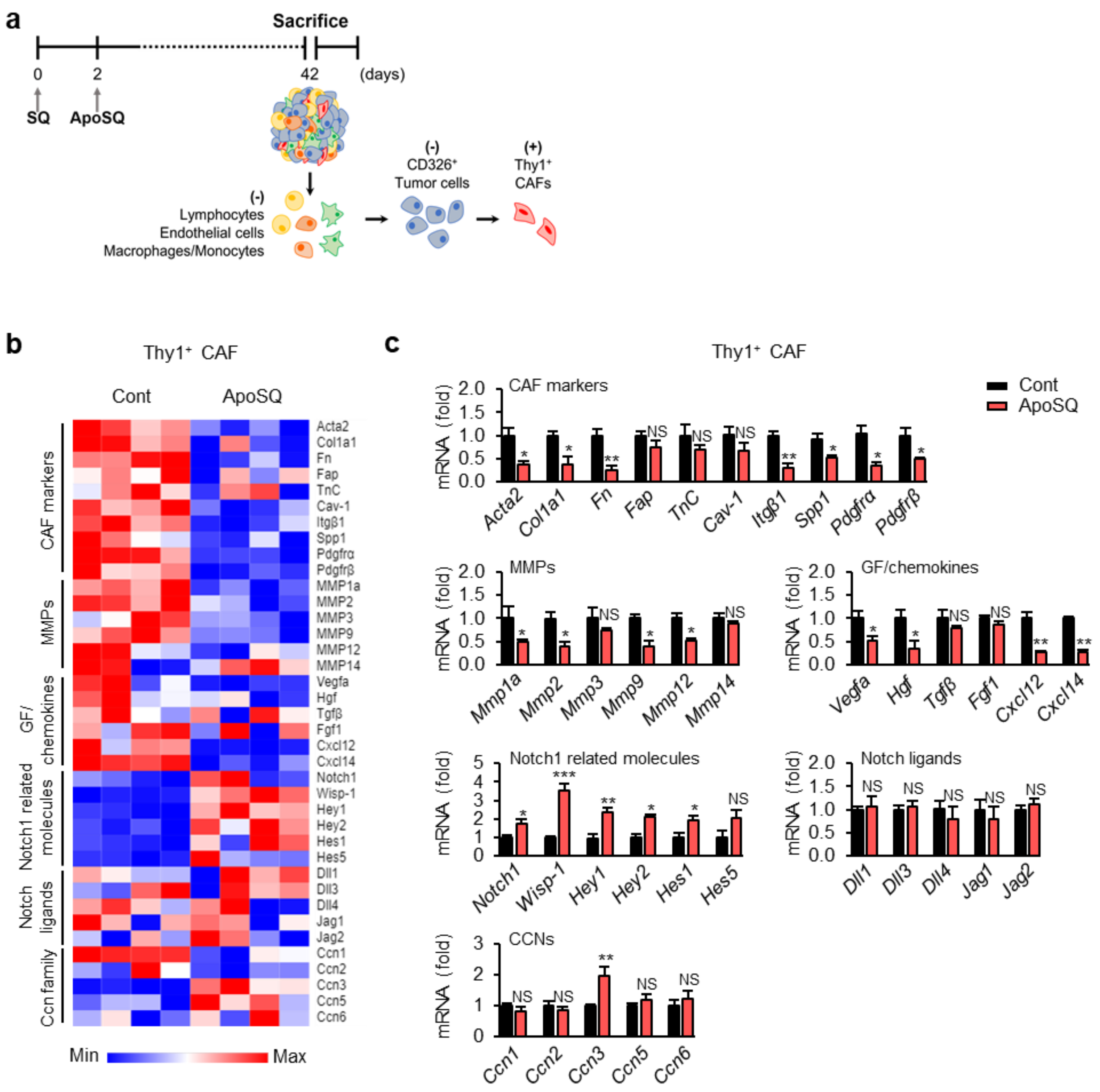

Supplementary Fig. S11. Injection of apoptotic 344SQ cells suppresses mRNA expression of CAFs activation markers, but enhances Notch1 downstream target genes in isolated Thy1⁺ CAFs. (a) A schematic of the experimental design. Apoptotic 344SQ cells (ApoSQ) were subcutaneously injected in the skin lesion 2 days after subcutaneous injection of 344SQ cells into syngeneic (129/Sv) mice (n=12 per group). Mice were necropsied 6 weeks after 344SQ cell injection. (**b**) Heatmap showing qRT-PCR data of differentially expressed genes for CAF markers, matrix metalloproteases (Mmps), growth factors (GF)/chemokines, and Notch1 related molecules with CCN family in isolated Thy1⁺ CAFs from primary tumors between control and ApoSQ group. Expression levels of each gene in a single sample is depicted according to the color scale. Red: high expression; blue: low expression. (**c**) qRT-PCR analysis of CAFs markers, MMPs,

GF/chemokines, Notch1 related molecules, and CCN family in isolated Thy1$^+$ CAFs. Data are from four replicates per condition with cells pooled from three mice per replicate. NS, not significant; *P < 0.05, **P < 0.01, ***P < 0.001, Kruskal-Wallis tests with Dunn's post hoc test. Data represent as means ± standard error (**c**).

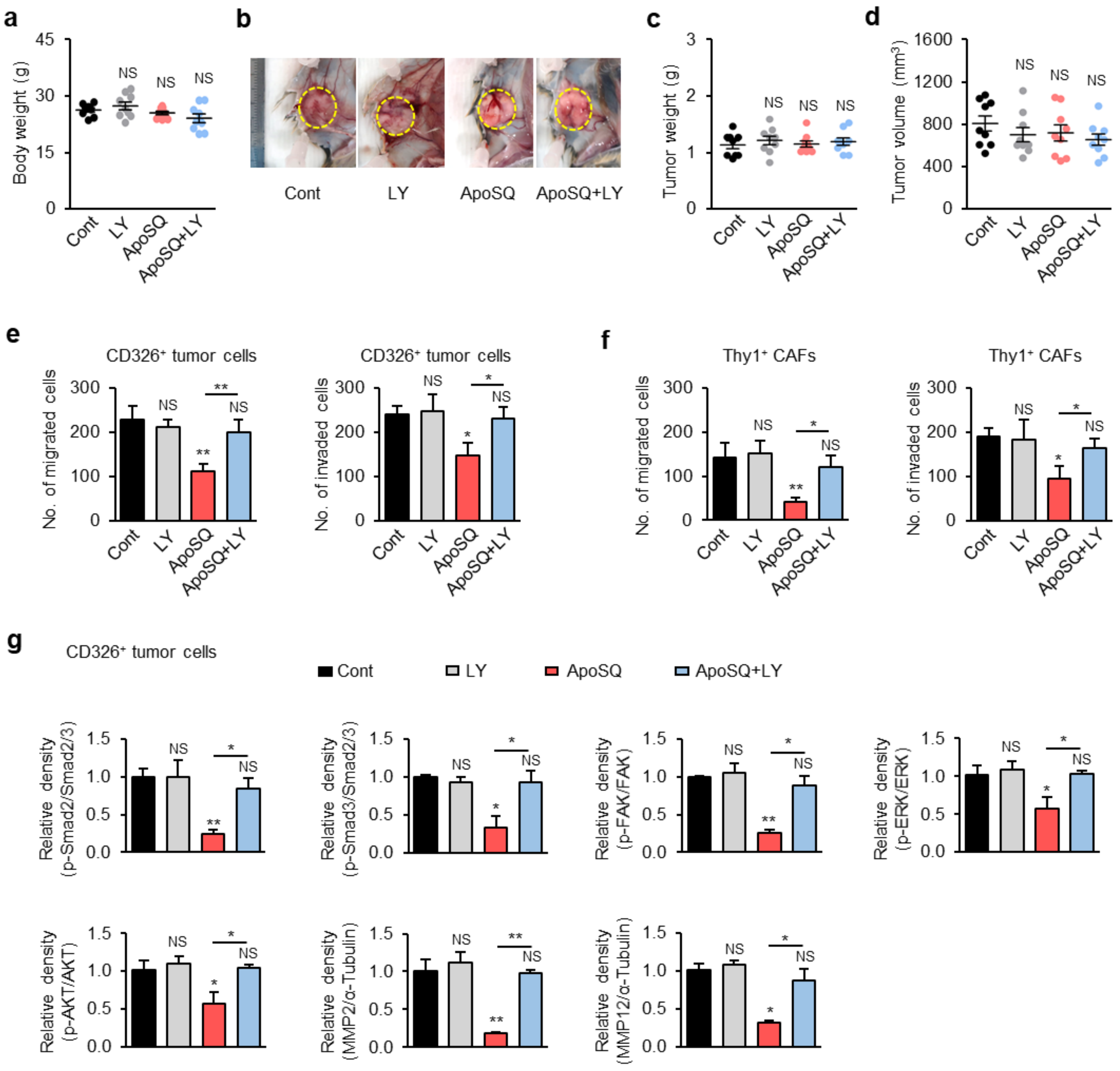

Supplementary Fig. S12. Inhibition of Notch1 signaling reverses the inhibition of migration, invasion and signaling pathways *in vivo*. ApoSQ were subcutaneously injected in the skin lesion 2 days after subcutaneous injection of 344SQ cells into syngeneic (129/Sv) mice (n=9 per group). Where indicated, LY3039478 (8 mg/kg/day, i.p.) or its vehicle (Veh; 15% sugar gel) was orally administered three times a week for 6 weeks starting the day before injection of ApoSQ (n=9 per group). Mice were necropsied 6 weeks after 344SQ cell injection. (**a**) Scatter plots of body

weight. (**b**) Representative images of primary tumors (yellow color dotted circle). Scatter plots of primary tumor weight (**c**) and tumor volume (**d**). (**a**, **c**, **d**) Data are shown as mean ± standard error (**a**, **c**, **d**). NS, not significant; Kruskal-Wallis tests with Dunn's post hoc test. Quantification of migrated and invaded CD326$^+$ tumor cells (**e**) and Thy1$^+$ CAFs (**f**) isolated from primary tumors. (**g**) Densitometric analysis of the relative abundances of the indicated proteins in isolated CD326$^+$ tumor cells. NS, not significant; *$P < 0.05$, **$P < 0.01$, Kruskal-Wallis tests with Dunn's post hoc test. Data from three replicates per condition with cells pooled from three mice per replicate. Data are represented from three independent experiments (mean ± standard error, **e**-**g**)

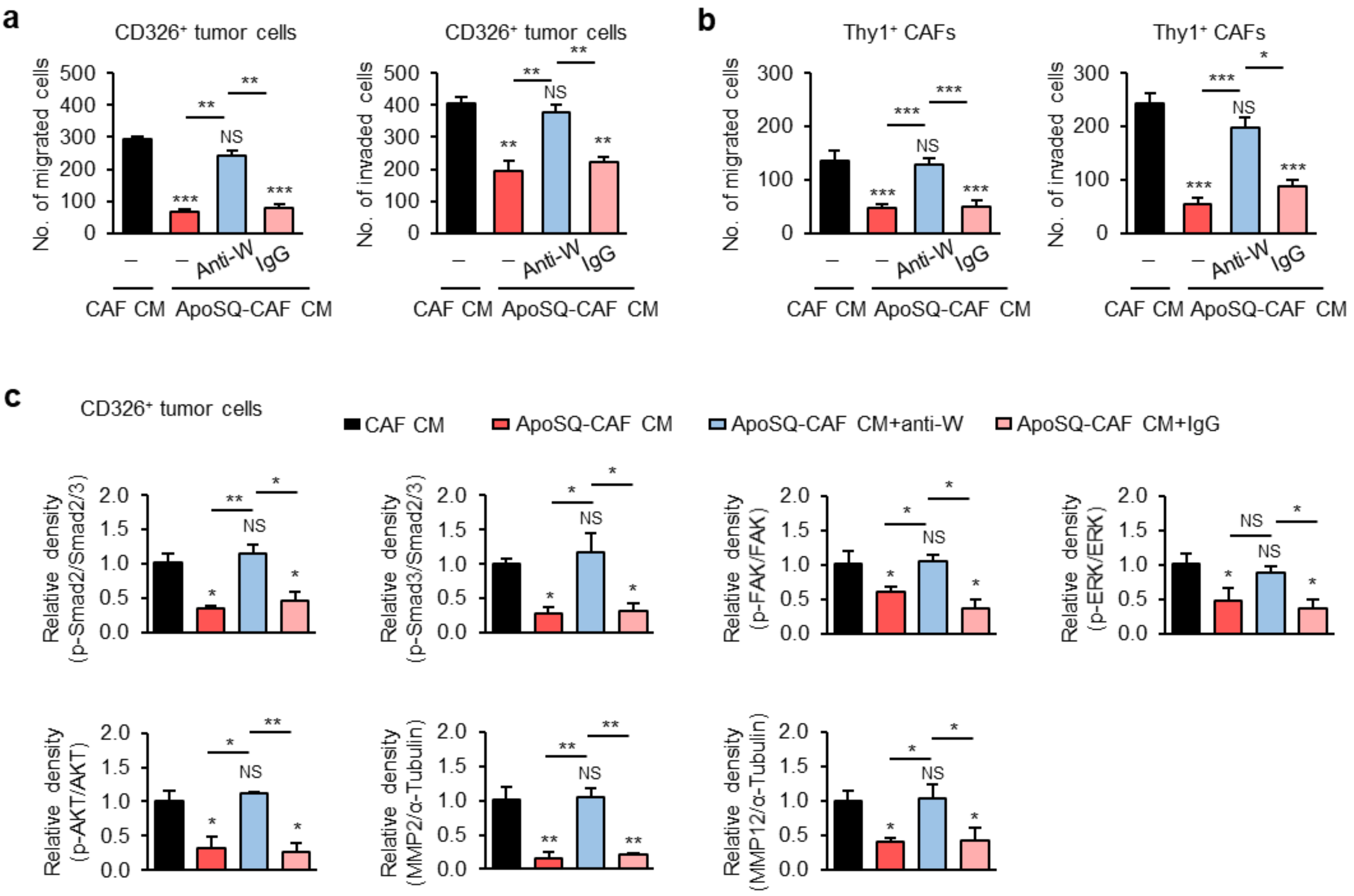

Supplementary Fig. S13. ApoSQ-CAF CM inhibits migration, invasion and signaling pathways via WISP-1 *in vivo*. CAF CM, ApoSQ-CAF CM, ApoSQ-CAF CM + anti-WISP-1, or ApoSQ-CAF CM + IgG was intratumorally injected three times a week for 6 weeks starting 2 days after subcutaneous injection of 344SQ cells into syngeneic (129/Sv) mice (n=8 per group). Mice were necropsied 6 weeks later. Quantification of migrated and invaded CD326$^+$ tumor cells (**a**) and Thy1$^+$ CAFs (**b**) isolated from primary tumors. (**c**) Densitometric analysis of the relative abundances of the indicated proteins in CD326$^+$ tumor cells. NS, not significant; *$P < 0.05$, **$P < 0.01$, ***$P < 0.001$, Kruskal-Wallis tests with Dunn's post hoc test. Data from n=3 replicates per condition with cells pooled from 2 or 3 mice per replicate. Data are represented as means ± standard error.

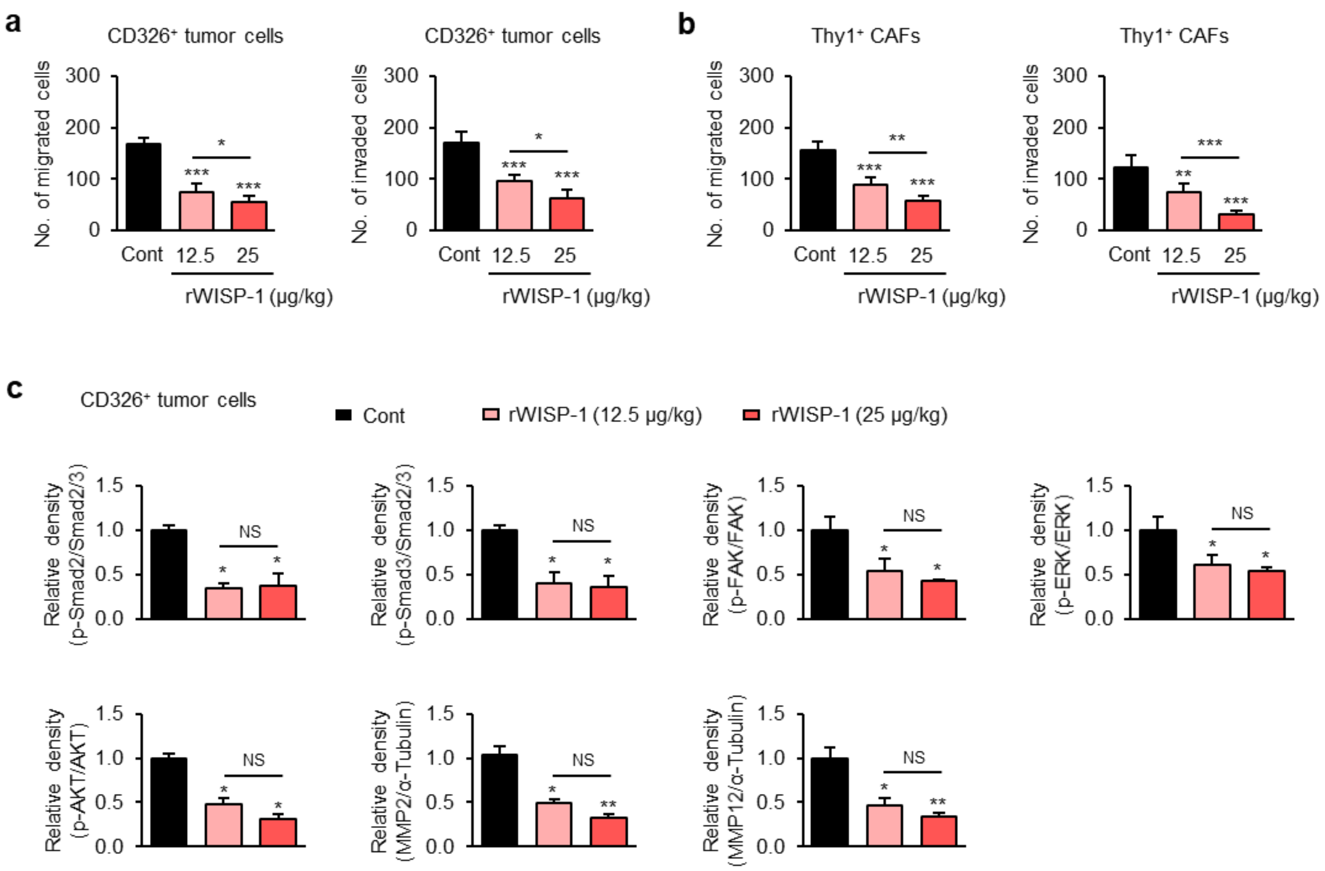

Supplementary Fig. S14. Injection of recombinant WISP-1 inhibits migration, invasion and signaling pathways. rWISP-1 (12.5 or 25 μg/kg) was intratumorally injected three times a week for 6 weeks starting 2 days after subcutaneous injection of 344SQ cells into syngeneic (129/Sν) mice (n=8 per group). Mice were necropsied 6 weeks later. Quantification of migrated and invaded CD326$^+$ tumor cells (**a**) and Thy1$^+$ CAFs (**b**) isolated from primary tumors. (**c**) Densitometric analysis of the relative abundances of the indicated proteins in CD326$^+$ tumor cells. NS, not significant; **P* < 0.05, ***P* < 0.01, ****P* < 0.001, Kruskal-Wallis tests with Dunn's post hoc test. Data from n=3 replicates per condition with cells pooled from 2 or 3 mice per replicate. Data are represented as means ± standard error.

Supplementary Table S1. List of antibodies used this study.

Antigen	Vendor	Cat.No	Source	Species cross-reactivity	Application	Dilution
p-Smad2	Cell Signaling	3108	Rabbit monoclonal	H, M, R, Mi	IB	1:1000
p-Smad3	Cell Signaling	9520	Rabbit monoclonal	H, M, R	IB	1:1000
p-AKT	Cell Signaling	4060	Rabbit monoclonal	H, M, R, Hm, Mk, Dm, Z, B	IB	1:1000
p-FAK	Cell Signaling	3283	Rabbit polyclonal	H, M, R, Hm, Pg	IB	1:1000
p-Src	Cell Signaling	2101	Rabbit polyclonal	H, M, R	IB	1:1000
p-ERK	Cell Signaling	9101	Rabbit polyclonal	B, Ce, Dm, Hm, H, Mi, Mk, M	IB	1:1000
p-p38	Santa Cruz	sc-17852-R	Rabbit polyclonal	H, M, R	IB	1:1000
Smad2/3	Cell Signaling	3102	Rabbit polyclonal	H, M, R, Mk	IB	1:1000
Akt	Cell Signaling	4691	Rabbit monoclonal	H, M, R, Mk, Dm	IB	1:1000
FAK	Cell Signaling	3285	Rabbit polyclonal	H, M, R, Hm, B, Pg	IB	1:1000
Src	Santa Cruz	sc-19	Rabbit polyclonal	H, M, R	IB	1:1000
ERK	Santa Cruz	sc-93-G	Goat polyclonal	H, M, R, C, F	IB	1:1000
p38	Santa Cruz	sc-535-G	Goat polyclonal	H, M, R	IB	1:1000
MMP2	Cell Signaling	#87809	Rabbit monoclonal	H, M	IB	1:1000
MMP12	Santa Cruz	sc-390863	Mouse monoclonal	H, M, R	IB	1:1000
α-SMA	Abcam	ab7817	Mouse monoclonal	H, M, R, Rb, Pg	IB, IF, IHC	1:1000<br>1:200
Col1α	GeneTex	GRX82721	Rabbit polyclonal	H, M, R	IB	1:1000
FN	Abcam	ab2413	Rabbit polyclonal	H, M	IB	1:1000
LIF	Abcam	ab113262	Rabbit polyclonal	H, M, R	IB	1:1000
WISP-1	Abcam	ab178547	Rabbit polyclonal	H, M, R	IB, IF	1:1000
	R&D Systems	AF1680	Sheep polyclonal	M	IF, IHC	1:200
NICD1	Cell Signaling	#4147	Rabbit monoclonal	H, M, R	IB, IF, IHC	1:1000<br>1:200
	Abcam	ab8925	Rabbit	M, H, R	IHC-P, IB	1:1000
Notch-1	Santa Cruz	sc-373891	Mouse monoclonal	H, M, R	IHC	1: 100
Hes1	Abcam	ab71559	Rabbit polyclonal	H, M, R	IB	1:1000
DLL1	Invitrogen	PA5-19106	Goat polyclonal	H, M, R	IB, FACS<br>Neutralizing	1:1000<br>1:100<br>20 μg/ml
DLL3	Invitrogen	PA5-26336	Rabbit polyclonal	H, M, R	IB, FACS	1:1000<br>1:100
DLL4	Invitrogen	PA5-85931	Rabbit polyclonal	H, M	IB, FACS	1:1000<br>1:100
Jag1	Cell Signaling	2620	Rabbit monoclonal	H, M	IB, FACS	1:1000<br>1:100
Jag2	Santa Cruz	sc-515725	Mouse monoclonal	H, M, R	IB, FACS	1:1000<br>1:100
β-Actin	Santa Cruz	sc-69879	Mouse monoclonal	Broad species	IB	1:1000
α-Tubulin	SIGMA	T5168	Mouse monoclonal	M, R	IB	1:3000
BAI1	R&D Systems	AF4969	Sheep polyclonal	H, M	Neutralizing	8 μg/ml
WISP-1	R&D Systems	MAB1680	Rat monoclonal	M	Neutralizing	10 μg/ml
IgG	R&D Systems	MAB0061	Rat monoclonal	M	Neutralizing	10 μg/ml
IgG	eBioscience	14-4888	Armenian hamster monoclonal	Broad species	Neutralizing	
integrin αv	eBioscience	14-0512	Rat monoclonal	H,M	Neutralizing	
integrin α5	eBioscience	14-0493	Armenian hamster monoclonal	M,R	Neutralizing	10 μg/ml (CAF)
integrin β1	eBioscience	16-0291	Armenian hamster monoclonal	M,R	Neutralizing	3 μg/ml (344SQ)
integrin β3	eBioscience	11-0611	Armenian hamster monoclonal	M, R	Neutralizing	
integrin β5	eBioscience	14-0497	Mouse monoclonal	Hm,H,M	Neutralizing	
CD31	Abcam	ab7388	Rat monoclonal	M	Cell sorting	
CD45	Abcam	ab25386	Rat monoclonal	M	Cell sorting	
CD68	Abcam	ab53444	Rat monoclonal	M	Cell sorting	
CD326	BD Bioscience	552370	Rat monoclonal	M	Cell sorting	
Mouse IgG (HRP)	GeneTex	GTX213111	Goat	Not applicable	IB	1:5000
Rabbit IgG (HRP)	GeneTex	GTX213110	Goat	Not applicable	IB	1:5000
Goat IgG (HRP)	Santa Cruz	sc-2354	Mouse	Not applicable	IB	1:5000
Mouse IgG (Alexa 488)	Thermo Fisher Scientific	A11029	Goat	Not applicable	FACS, IHC	1:100<br>1:400
Mouse IgG (Alexa 594)	Thermo Fisher Scientific	A11032	Goat	Not applicable	IHC	1:400
Rabbit IgG (Alexa 488)	Thermo Fisher Scientific	A11034	Goat	Not applicable	FACS, IF, IHC	1:100<br>1:400
Rabbit IgG (Alexa 594)	Thermo Fisher Scientific	A11012	Goat	Not applicable	FACS. IF, IHC	1:100<br>1:400
Goat IgG (Alexa 488)	Thermo Fisher Scientific	A11055	Donkey	Not applicable	FACS	1:100
Goat IgG (Alexa 647)	Thermo Fisher Scientific	A21447	Donkey	Not applicable	FACS	1:100
Sheep IgG (Alexa 594)	Thermo Fisher Scientific	A11016	Donkey	Not applicable	IF, IHC	1:400

Abbreviation: IB-Immunoblot, IHC-Immunohistochemistry, IHC-P-Immunohisrochemistry-paraffin, IF-Immunofluorescence, H-human, M-mouse, R-rat, Rb-rabbit, P-pig, Mi-mink, Hm-hamster, Mk-monkey, Dm-drosophila melanogaster, Z-zebrafish, B-Bovine, Pg-pig, Ce-caenorhabditis elegans, F-frog

Supplementary Table S2. qPCR primers used in this study.

murine gene	forward (5' → 3')	reverse (5' → 3')
Acta2	CCCAGATTATGTTTGAGACCTTC	ATCTCCAGAGTCCAGCACAATAC
Cav-1	CACACCAAGGAGATTGACCTGG	CCTTCCAGATGCCGTCGAAACT
Ccn1	TTTACAGTTGGGCTGGAAGC	CACCGCTCTGAAAGGGATCT
Ccn2	GCTTGGCGATTTTAGGTGTC	CAGACTGGAGAAGCAGAGCC
Ccn3	GTCACCAACAGGAATCGCCAGT	GTAGGTGGATGGCTTTCAGGGA
Ccn5	TGTGTGACCAGGCAGTGATGCA	CAGGCTGTGCTCCAGTTTGGAC
Ccn6	CACCTGTAACGAAGCCGAGATC	ACTCACATCCAACTGCCACAAGA
Col1a1	CAAGAAGACATCCCTGAAGTC	ACAGTCCAGTTCTTCATTGC
Cxcl12	TGCATCAGTGACGGTAAACCA	CACAGTTTGGAGTGTTGAGGAT
Cxcl14	TACCCACACTGCGAGGAGAAGA	CGCTTCTCGTTCCAGGCATTGT
Dll1	CCGGCTGAAGCTACAGAAAC	AGCCCCAATGATGCTAACAG
Dll3	CTGGTGTCTTCGAGCTACAAAT	TGCTCCGTATAGACCGGGAC
Dll4	CCTCTCGAACTTGGACTTGC	TGGAAATACAGATGCCCACA
Fap	GTCACCTGATCGGCAATTTGT	CCCCATTCTGAAGGTCGTAGAT
Fgf1	CCAAGGAAACGTCCACAGTCAG	ACGGCTGAAGACATCCTGTCTC
Fn	CACGATGCGGGTCACTTG	CTGCAACGTCCTCTTCATTCTTC
Hes1	CTACCCCAGCCAGTGTCAAC	CCTTCGCCTCTTCTCCATGA
Hes5	ATCAACAGCAGCATAGAGCAG	CGAAGGCTTTGCTGTGTTTCA
Hey1	ACATCGTCCCAGGTTTTGGC	GCTTCTCAAAGGCACTGGGT
Hey2	TCCAGGCTACAGGGGGTAAA	CAAGGCCTTCCACTGAGCTT
Hgf	CCTGACACCACTTGGGAGTA	CTTCTCCTTGGCCTTGAATG
Hprt	CCAGTGTCAATTATATCTTCAAC	CAGACTGAAGAGCTACTGTAATG
Itgβ1	CTCCAGAAGGTGGCTTTGATGC	GTGAAACCCAGCATCCGTGGAA
Jag1	GGAAGTGGAGGAGGATGACA	GTCCAGTTCGGGTGTTTTGT
Jag2	TCCGAGTACGCTGTGATGAG	GGCTTCTTTGCATTCTTTGC
Mmp1a	AGGAAGGCGATATTGTGCTCTCC	TGGCTGGAAAGTGTGAGCAAGC
Mmp2	CAAGGATGGACTCCTGGCACAT	TACTCGCCATCAGCGTTCCCAT
Mmp3	CTCTGGAACCTGAGACATCACC	AGGAGTCCTGAGAGATTTGCGC
Mmp9	TGCCCAGCGACCACAACTC	CGGACCCGAAGC GGACATT
Mmp12	CACACTTCCCAGGAATCAAGCC	TTTGGTGACACGACGGAACAGG
Mmp14	GTGAGCGTTGTGTGTGGGTA	CCCAAGGCA GCAACTTCAG
Notch1	GCCGCAAGAGGCTTGAGAT	GGAGTCCTGGCATCGTTGG
Pdgfrα	CTCTTGGAGATAGACTCCGTAGG	ACTTCTCTTCCTGCGAATGG
Pdgfrβ	TGGCCTCTGAGGACTAAAGC	AACAGAAGACAGCGAGGTGG
Spp1	GCTTGGCTTATGGACTGAGGTC	CCTTAGACTCACCGCTCTTCATG
Tgfβ	TGCCGTACAACTCCAGTGAC	TGGAGCAACATGTGGAACTC
Tnc	CCATCAGTACCACGGCTACC	CCCTTCATCAGCAGTCCAGG
Vegfa	GTACCTCCACCATGCCAAGT	TCACATCTGCAAGTACGTTCG
Wisp-1(ccn4)	CAATAGGAGTGTGTGCACAGGT	TACCTGCAGTTGGGTTGGAAG
